
JOURNAL INFORMATION
==============================
NLM Title Abbreviation: J Int AIDS Soc
Journal ID: J Int AIDS Soc
NLM Journal ID: 101478566
Journal ID: JIAS

Journal of the International AIDS Society

EISSN: 1758-2652
Publisher: Wiley

ARTICLE INFORMATION
==============================
PMCID: PMC3499904
DOI: 10.7448/IAS.15.5.18443
Article ID: 18443
Article version: 1
Subjects: AIDS2012 Abstract Supplement

Track E Implementation Science, Health Systems and Economics

Electronic publication date: 2012 Oct 22
Publication date: 2012
Volume: 15
Issue: Suppl 3
Electronic Location ID: 18443
Copyright: © 2012 Track E Implementation Science, Health Systems and Economics
Copyright year: 2012
License: This is an open-access article distributed under the terms of the Creative Commons Attribution License, which permits unrestricted use, distribution, and reproduction in any medium, provided the original work is properly cited.
License URL: https://creativecommons.org/licenses/by/2.0/

==============================
JOURNAL INFORMATION
==============================
NLM Title Abbreviation: J Int AIDS Soc
Journal ID: J Int AIDS Soc
NLM Journal ID: 101478566
Journal ID: JIAS

Journal of the International AIDS Society

EISSN: 1758-2652
Publisher: Wiley

ARTICLE INFORMATION
==============================
Article ID: 18443-01
Subjects: E1 - Integrating HIV inpatient and outpatient services, HIV-TB, HIV-STI, non-communicable disorders and other relevant diseases

MOAE0102: Family health days: an innovative approach to providing integrated health services for HIV and non-communicable diseases among adults and children in hard-to-reach areas of Lesotho

Tiam A. 1 §
Oyebanji O. 1
Nkonyana J. 2
Ahimbisibwe A. 1
Putsoane M. 1
Mokone M. 1
Nyabela M. 1
Isavwa A. 1
Tsoeu M. 1
Foso M. 1
Buhendwa L. 1
1 Elizabeth Glaser Pediatric AIDS Foundation (EGPAF), Clinical Services, Maseru, Lesotho
2 MOHSW-Lesotho, DDC, Maseru, Lesotho
§ Presenting author email: atiam@pedaids.org
Electronic publication date: 2012 Oct 22
Publication date: 2012
Volume: 15
Issue: Suppl 3
Electronic Location ID: 18443-01
Copyright: © 2012 A. Tiam et al.
Copyright year: 2012
License: This is an open-access article distributed under the terms of the Creative Commons Attribution License, which permits unrestricted use, distribution, and reproduction in any medium, provided the original work is properly cited.
License URL: http://creativecommons.org/licenses/by/2.0/

JOURNAL INFORMATION
==============================
NLM Title Abbreviation: J Int AIDS Soc
Journal ID: J Int AIDS Soc
NLM Journal ID: 101478566
Journal ID: JIAS

Journal of the International AIDS Society

EISSN: 1758-2652
Publisher: Wiley

ARTICLE INFORMATION
==============================
Article ID: 18443-02
Subjects: E1 - Integrating HIV inpatient and outpatient services, HIV-TB, HIV-STI, non-communicable disorders and other relevant diseases

MOAE0103: Integrated community HIV testing campaigns: leveraging HIV infrastructure for non-communicable diseases

Chamie G. 1 2 §
Kwarisiima D. 3
Clark T. 1 2
Kabami J. 2
Jain V. 1 2
Geng E. 1 2
Petersen M. 4
Thirumurthy H. 5
Kamya M. 6
Havlir D.V. 1 2
Charlebois E. 2 7
SEARCH Consortium
1 University of California, San Francisco, Division of HIV/AIDS, San Francisco General Hospital, San Francisco, United States
2 Makerere University-University of California, San Francisco (MU-UCSF) Research Collaboration, Kampala, Uganda
3 Mulago-Mbarara Teaching Hospitals' Joint AIDS Program (MJAP), Kampala, Uganda
4 School of Public Health, University of California, Berkeley, United States
5 Gillings School of Global Public Health, University of North Carolina, Chapel Hill, United States
6 Department of Medicine, School of Medicine, Makerere University College of Health Sciences, Kampala, Uganda
7 Department of Medicine, University of California, Center for AIDS Prevention Studies, San Francisco, United States
§ Presenting author email: gabriel.chamie@ucsf.edu
Electronic publication date: 2012 Oct 22
Publication date: 2012
Volume: 15
Issue: Suppl 3
Electronic Location ID: 18443-02
Copyright: © 2012 G. Chamie et al.
Copyright year: 2012
License: This is an open-access article distributed under the terms of the Creative Commons Attribution License, which permits unrestricted use, distribution, and reproduction in any medium, provided the original work is properly cited.
License URL: http://creativecommons.org/licenses/by/2.0/

JOURNAL INFORMATION
==============================
NLM Title Abbreviation: J Int AIDS Soc
Journal ID: J Int AIDS Soc
NLM Journal ID: 101478566
Journal ID: JIAS

Journal of the International AIDS Society

EISSN: 1758-2652
Publisher: Wiley

ARTICLE INFORMATION
==============================
Article ID: 18443-03
Subjects: E3 - Effect of HIV funding/programming on sexual and reproductive health (SRH) services

WEPDE0101: Managing change in performance-based project funding: a case study of PPFN Rapid Emergency Scale Up Plan to increase HCT/FP/STIs integrated services

Hounkanrin G. 1
Melkamu Y. 1
Ibrahim I.M. 2 §
Ogo I. 2
Opeyemi O. 2
Toki D. 2
1 International Planned Parenthood Federation, Africa Region, Nairobi, Kenya
2 Planned Parenthood Federation of Nigeria (PPFN), Abuja, Nigeria
§ Presenting author email: imibrahim@ppfn.org
Electronic publication date: 2012 Oct 22
Publication date: 2012
Volume: 15
Issue: Suppl 3
Electronic Location ID: 18443-03
Copyright: © 2012 I.M. Ibrahim et al.
Copyright year: 2012
License: This is an open-access article distributed under the terms of the Creative Commons Attribution License, which permits unrestricted use, distribution, and reproduction in any medium, provided the original work is properly cited.
License URL: http://creativecommons.org/licenses/by/2.0/

JOURNAL INFORMATION
==============================
NLM Title Abbreviation: J Int AIDS Soc
Journal ID: J Int AIDS Soc
NLM Journal ID: 101478566
Journal ID: JIAS

Journal of the International AIDS Society

EISSN: 1758-2652
Publisher: Wiley

ARTICLE INFORMATION
==============================
Article ID: 18443-04
Subjects: E4 - Integration of SRH and HIV services: Delivery models and costs

WEPDE0102: Reaching key populations in SRH/HIV integration: recommendations from a global intervention review to identify strategies to increase the responsiveness and relevance of integrated programming to the sexual and reproductive health and rights (SRHR) needs of high-risk groups, including sex workers, MSM, transgenders, IDUs and PLHIV

Grote S. 1 §
Middleton-Lee S. 2
Robertson J. 1
Mehta S. 1
1 India HIV/AIDS Alliance, New Delhi, India
2 Independent Consultant, Brighton, United Kingdom
§ Presenting author email: sunitagrote@gmail.com
Electronic publication date: 2012 Oct 22
Publication date: 2012
Volume: 15
Issue: Suppl 3
Electronic Location ID: 18443-04
Copyright: © 2012 S. Grote et al.
Copyright year: 2012
License: This is an open-access article distributed under the terms of the Creative Commons Attribution License, which permits unrestricted use, distribution, and reproduction in any medium, provided the original work is properly cited.
License URL: http://creativecommons.org/licenses/by/2.0/

JOURNAL INFORMATION
==============================
NLM Title Abbreviation: J Int AIDS Soc
Journal ID: J Int AIDS Soc
NLM Journal ID: 101478566
Journal ID: JIAS

Journal of the International AIDS Society

EISSN: 1758-2652
Publisher: Wiley

ARTICLE INFORMATION
==============================
Article ID: 18443-05
Subjects: E8 - Task-shifting

MOPDE0103: Implementation of VMMC efficiency elements in four sub-Saharan countries: service delivery methods and provider attitudes

Bertrand J. 1 §
Rech D. 2
Njeuhmeli E. 3
Castor D. 3
Frade S. 2
Loolpapit M. 4
Machaku M. 5
Mavhu W. 6
Perry L. 1
1 Tulane School of Public Health and Tropical Medicine, Tulane SPHTM, New Orleans, United States
2 CHAPS, Johannesburg, South Africa
3 USAID, Washington, United States
4 FHI360/Kenya, Nairobi, Kenya
5 Jhpiego/Tanzania, Dar es Salaam, United Republic of Tanzania
6 ZAPP, Harare, Zimbabwe
§ Presenting author email: bertrand@tulane.edu
Electronic publication date: 2012 Oct 22
Publication date: 2012
Volume: 15
Issue: Suppl 3
Electronic Location ID: 18443-05
Copyright: © 2012 J. Bertrand et al.
Copyright year: 2012
License: This is an open-access article distributed under the terms of the Creative Commons Attribution License, which permits unrestricted use, distribution, and reproduction in any medium, provided the original work is properly cited.
License URL: http://creativecommons.org/licenses/by/2.0/

JOURNAL INFORMATION
==============================
NLM Title Abbreviation: J Int AIDS Soc
Journal ID: J Int AIDS Soc
NLM Journal ID: 101478566
Journal ID: JIAS

Journal of the International AIDS Society

EISSN: 1758-2652
Publisher: Wiley

ARTICLE INFORMATION
==============================
Article ID: 18443-06
Subjects: E9 - Models of delivery, including promotion of acceptance, uptake and costs

MOPDE0102: Determinants of VMMC provider burnout in four sub-Saharan countries

Bertrand J. 1
Rech D. 2 §
Njeuhmeli E. 3
Castor D. 3
Frade S. 2
Loolpapit M. 4
Machaku M. 5
Mavhu W. 6
Perry L. 1
1 Tulane SPHTM, Global Health Systems and Development, New Orleans, United States
2 CHAPS, Johannesburg, South Africa
3 USAID, Washington, United States
4 FHI360/Kenya, Nairobi, Kenya
5 Jhpiego/Tanzania, Dar es Salaam, United Republic of Tanzania
6 ZAPP, Harare, Zimbabwe
§ Presenting author email: dino@chaps.org.za
Electronic publication date: 2012 Oct 22
Publication date: 2012
Volume: 15
Issue: Suppl 3
Electronic Location ID: 18443-06
Copyright: © 2012 D. Rech et al.
Copyright year: 2012
License: This is an open-access article distributed under the terms of the Creative Commons Attribution License, which permits unrestricted use, distribution, and reproduction in any medium, provided the original work is properly cited.
License URL: http://creativecommons.org/licenses/by/2.0/

JOURNAL INFORMATION
==============================
NLM Title Abbreviation: J Int AIDS Soc
Journal ID: J Int AIDS Soc
NLM Journal ID: 101478566
Journal ID: JIAS

Journal of the International AIDS Society

EISSN: 1758-2652
Publisher: Wiley

ARTICLE INFORMATION
==============================
Article ID: 18443-07
Subjects: E9 - Models of delivery, including promotion of acceptance, uptake and costs

MOPDE0106: A comparative analysis of two high-volume male medical circumcision (MMC) operational models with similar service delivery outcomes in different settings within Gauteng and KwaZulu-Natal provinces in South Africa: urban Centre for HIV/AIDS Prevention Studies (CHAPS) versus rural-SACTWU Worker Health Program (SWHP)

Soboil N. 1 §
Cockburn J. 1
Rech D. 2
Taljaard D. 2
1 SACTWU, Worker Health Program, Cape Town, South Africa
2 CHAPS, Johannesburg, South Africa
§ Presenting author email: nikki@swtzn.co.za
Electronic publication date: 2012 Oct 22
Publication date: 2012
Volume: 15
Issue: Suppl 3
Electronic Location ID: 18443-07
Copyright: © 2012 N. Soboil et al.
Copyright year: 2012
License: This is an open-access article distributed under the terms of the Creative Commons Attribution License, which permits unrestricted use, distribution, and reproduction in any medium, provided the original work is properly cited.
License URL: http://creativecommons.org/licenses/by/2.0/

JOURNAL INFORMATION
==============================
NLM Title Abbreviation: J Int AIDS Soc
Journal ID: J Int AIDS Soc
NLM Journal ID: 101478566
Journal ID: JIAS

Journal of the International AIDS Society

EISSN: 1758-2652
Publisher: Wiley

ARTICLE INFORMATION
==============================
Article ID: 18443-08
Subjects: E9 - Models of delivery, including promotion of acceptance, uptake and costs

MOPDE0107: We too are shareholders: why women must be meaningfully involved in the rollout of medical male circumcision in Africa

Agot K. 1 §
Ohaga S. 1
Ayieko B. 1
Omanga E. 1
Kabare M. 1
1 Impact Research & Development Organization, Kisumu, Kenya
§ Presenting author email: kawango@impact-rdo.org
Electronic publication date: 2012 Oct 22
Publication date: 2012
Volume: 15
Issue: Suppl 3
Electronic Location ID: 18443-08
Copyright: © 2012 K. Agot et al.
Copyright year: 2012
License: This is an open-access article distributed under the terms of the Creative Commons Attribution License, which permits unrestricted use, distribution, and reproduction in any medium, provided the original work is properly cited.
License URL: http://creativecommons.org/licenses/by/2.0/

JOURNAL INFORMATION
==============================
NLM Title Abbreviation: J Int AIDS Soc
Journal ID: J Int AIDS Soc
NLM Journal ID: 101478566
Journal ID: JIAS

Journal of the International AIDS Society

EISSN: 1758-2652
Publisher: Wiley

ARTICLE INFORMATION
==============================
Article ID: 18443-09
Subjects: E15 - Managing HIV supply chain challenges with limited resources

THPDE0101: Lessons from the rapid response of Namibia's supply chain when antiretroviral treatment guidelines changed to tenofovir-based first-line regimen

Lates J. 1 §
Ongeri B. 2
Wolde A. 2
Sagwa E. 2
Mabirizi D. 2
1 Division: Pharmaceutical Services, Ministry of Health and Social Services, Windhoek, Namibia
2 Supply Chain Management System (SCMS), Windhoek, Namibia
§ Presenting author email: jennie_lates@yahoo.co.uk
Electronic publication date: 2012 Oct 22
Publication date: 2012
Volume: 15
Issue: Suppl 3
Electronic Location ID: 18443-09
Copyright: © 2012 J. Lates et al.
Copyright year: 2012
License: This is an open-access article distributed under the terms of the Creative Commons Attribution License, which permits unrestricted use, distribution, and reproduction in any medium, provided the original work is properly cited.
License URL: http://creativecommons.org/licenses/by/2.0/

JOURNAL INFORMATION
==============================
NLM Title Abbreviation: J Int AIDS Soc
Journal ID: J Int AIDS Soc
NLM Journal ID: 101478566
Journal ID: JIAS

Journal of the International AIDS Society

EISSN: 1758-2652
Publisher: Wiley

ARTICLE INFORMATION
==============================
Article ID: 18443-10
Subjects: E15 - Managing HIV supply chain challenges with limited resources

THPDE0102: Cote d'Ivoire in crisis: supply chain strategies averted treatment interruption

Touhon M. 1
Pringle A. 2
Sheridan C. 2
Evi J.B. 3 §
1 Supply Chain Management System (SCMS), Abidjan, Cote D'Ivoire
2 Supply Chain Management System (SCMS), Arlington, United States
3 MSH/SCMS, Abidjan, Cote D'Ivoire
§ Presenting author email: bevi@ci.pfscm.org
Electronic publication date: 2012 Oct 22
Publication date: 2012
Volume: 15
Issue: Suppl 3
Electronic Location ID: 18443-10
Copyright: © 2012 J. B. Evi et al.
Copyright year: 2012
License: This is an open-access article distributed under the terms of the Creative Commons Attribution License, which permits unrestricted use, distribution, and reproduction in any medium, provided the original work is properly cited.
License URL: http://creativecommons.org/licenses/by/2.0/

JOURNAL INFORMATION
==============================
NLM Title Abbreviation: J Int AIDS Soc
Journal ID: J Int AIDS Soc
NLM Journal ID: 101478566
Journal ID: JIAS

Journal of the International AIDS Society

EISSN: 1758-2652
Publisher: Wiley

ARTICLE INFORMATION
==============================
Article ID: 18443-11
Subjects: E15 - Managing HIV supply chain challenges with limited resources

THPDE0103: Use of simple, low cost innovations to improve availability of HIV-related commodities at public hospitals in Uganda

Kizito H. 1
Tugume G. 1
Jaramillo L. 2 §
1 USAID Strengthening Uganda's Systems for Treating AIDS Nationally (SUSTAIN) Project, University Research Co., LLC (URC), Kampala, Uganda
2 International Development Group, University Research Co., LLC (URC), Bethesda, United States
§ Presenting author email: ljaramillo@urc-chs.com
Electronic publication date: 2012 Oct 22
Publication date: 2012
Volume: 15
Issue: Suppl 3
Electronic Location ID: 18443-11
Copyright: © 2012 L. Jaramillo et al.
Copyright year: 2012
License: This is an open-access article distributed under the terms of the Creative Commons Attribution License, which permits unrestricted use, distribution, and reproduction in any medium, provided the original work is properly cited.
License URL: http://creativecommons.org/licenses/by/2.0/

JOURNAL INFORMATION
==============================
NLM Title Abbreviation: J Int AIDS Soc
Journal ID: J Int AIDS Soc
NLM Journal ID: 101478566
Journal ID: JIAS

Journal of the International AIDS Society

EISSN: 1758-2652
Publisher: Wiley

ARTICLE INFORMATION
==============================
Article ID: 18443-12
Subjects: E15 - Managing HIV supply chain challenges with limited resources

THPDE0105: Overcoming challenges in supply chain management amidst rapid scale-up of antiretroviral services

Mulema V.S. 1
Kabogoza S. 1 §
Tumuhairwe D. 1
Mumbe L. 2
Kimuli R. 1
Mugume A. 1
1 JSI Research & Training Institute, Inc. (JSI)/Strengthening TB and HIV&AIDS Responses in East Central Uganda (STAR-EC), Jinja, Uganda
2 Ministry of Health, AIDS Control Programme, Kampala, Uganda
§ Presenting author email: skabogoza@starecuganda.org
Electronic publication date: 2012 Oct 22
Publication date: 2012
Volume: 15
Issue: Suppl 3
Electronic Location ID: 18443-12
Copyright: © 2012 S. Kabogoza et al.
Copyright year: 2012
License: This is an open-access article distributed under the terms of the Creative Commons Attribution License, which permits unrestricted use, distribution, and reproduction in any medium, provided the original work is properly cited.
License URL: http://creativecommons.org/licenses/by/2.0/

JOURNAL INFORMATION
==============================
NLM Title Abbreviation: J Int AIDS Soc
Journal ID: J Int AIDS Soc
NLM Journal ID: 101478566
Journal ID: JIAS

Journal of the International AIDS Society

EISSN: 1758-2652
Publisher: Wiley

ARTICLE INFORMATION
==============================
Article ID: 18443-13
Subjects: E15 - Managing HIV supply chain challenges with limited resources

THPDE0106: Mind the gap: evaluating internal controls in HIV supply chains across sub-Saharan Africa

Kohler J. 1 §
Revathi A. 2
1 Deloitte Consulting LLP, Washington, United States
2 Deloitte Consulting, LLP, Arlington, United States
§ Presenting author email: julkohler@deloitte.com
Electronic publication date: 2012 Oct 22
Publication date: 2012
Volume: 15
Issue: Suppl 3
Electronic Location ID: 18443-13
Copyright: © 2012 J. Kohler et al.
Copyright year: 2012
License: This is an open-access article distributed under the terms of the Creative Commons Attribution License, which permits unrestricted use, distribution, and reproduction in any medium, provided the original work is properly cited.
License URL: http://creativecommons.org/licenses/by/2.0/

JOURNAL INFORMATION
==============================
NLM Title Abbreviation: J Int AIDS Soc
Journal ID: J Int AIDS Soc
NLM Journal ID: 101478566
Journal ID: JIAS

Journal of the International AIDS Society

EISSN: 1758-2652
Publisher: Wiley

ARTICLE INFORMATION
==============================
Article ID: 18443-14
Subjects: E17 - Developing/implementing chronic disease programmes in resource-limited settings

MOAE0104: Cardiovascular disease risk factor profiles of HIV-positive clients: findings from a pilot program to integrate CVD screening into HIV services at a secondary health facility in Kano, North-Western Nigeria

Gwarzo U. 1 §
Maji T. 1
Isa-Dutse S. 1
Ahmed Y. 1
Obayagbona K. 1
Okechukwu E. 2
Odafe S. 1
Khamofu H. 1
Torpey K. 1
Chabikuli O. 1
1 FHI360 (Family Health International), Abuja, Nigeria
2 USAID Nigeria, Abuja, Nigeria
§ Presenting author email: ugwarzo@ghain.org
Electronic publication date: 2012 Oct 22
Publication date: 2012
Volume: 15
Issue: Suppl 3
Electronic Location ID: 18443-14
Copyright: © 2012 U. Gwarzo et al.
Copyright year: 2012
License: This is an open-access article distributed under the terms of the Creative Commons Attribution License, which permits unrestricted use, distribution, and reproduction in any medium, provided the original work is properly cited.
License URL: http://creativecommons.org/licenses/by/2.0/

JOURNAL INFORMATION
==============================
NLM Title Abbreviation: J Int AIDS Soc
Journal ID: J Int AIDS Soc
NLM Journal ID: 101478566
Journal ID: JIAS

Journal of the International AIDS Society

EISSN: 1758-2652
Publisher: Wiley

ARTICLE INFORMATION
==============================
Article ID: 18443-15
Subjects: E17 - Developing/implementing chronic disease programmes in resource-limited settings

MOAE0105: Integrating cervical cancer prevention services into mobile HIV counseling and testing services to reach more women with life-saving cancer interventions

Mulenga E. 1
Chisoko C. 2
Mulenga Y. 1
Makoane C. 3
1 PCI, Lusaka, Zambia
2 Zambia Defence Force, Lusaka, Zambia
3 PCI, Washington, United States
Electronic publication date: 2012 Oct 22
Publication date: 2012
Volume: 15
Issue: Suppl 3
Electronic Location ID: 18443-15
Copyright: © 2012 E. Mulenga et al.
Copyright year: 2012
License: This is an open-access article distributed under the terms of the Creative Commons Attribution License, which permits unrestricted use, distribution, and reproduction in any medium, provided the original work is properly cited.
License URL: http://creativecommons.org/licenses/by/2.0/

JOURNAL INFORMATION
==============================
NLM Title Abbreviation: J Int AIDS Soc
Journal ID: J Int AIDS Soc
NLM Journal ID: 101478566
Journal ID: JIAS

Journal of the International AIDS Society

EISSN: 1758-2652
Publisher: Wiley

ARTICLE INFORMATION
==============================
Article ID: 18443-16
Subjects: E17 - Developing/implementing chronic disease programmes in resource-limited settings

MOAE0305: Sustainable financing for national responses through use of locally mobilised resources: a case of the AIDS levy in Zimbabwe

Yekeye R. 1
Manenji A. 2
Magure D.T. 1
1 National AIDS Council of ZImbabwe, Programmes, Harare, Zimbabwe
2 National AIDS Council of Zimbabwe, Finance, Harare, Zimbabwe
Electronic publication date: 2012 Oct 22
Publication date: 2012
Volume: 15
Issue: Suppl 3
Electronic Location ID: 18443-16
Copyright: © 2012 R. Yekeye et al.
Copyright year: 2012
License: This is an open-access article distributed under the terms of the Creative Commons Attribution License, which permits unrestricted use, distribution, and reproduction in any medium, provided the original work is properly cited.
License URL: http://creativecommons.org/licenses/by/2.0/

JOURNAL INFORMATION
==============================
NLM Title Abbreviation: J Int AIDS Soc
Journal ID: J Int AIDS Soc
NLM Journal ID: 101478566
Journal ID: JIAS

Journal of the International AIDS Society

EISSN: 1758-2652
Publisher: Wiley

ARTICLE INFORMATION
==============================
Article ID: 18443-17
Subjects: E21 - Using mobile phones/other methods to track patient and for programme data

WEAE0302: Monthly monitoring at a small NGO: mobile phones versus paper

Regan K. 1 2 §
Seo D. 1 3
Churchman E. 1 4
Kiwango J. 1
Gervas S. 1
Whiddon L. 1
1 Support for International Change (SIC), Arusha, United Republic of Tanzania
2 University of Maryland, College Park, Education Policy Studies, College Park, United States
3 University of Michigan, William Davidson Institute, Ann Arbor, United States
4 Emory University, Hubert Department of Global Health, Atlanta, United States
§ Presenting author email: kati@sichange.org
Electronic publication date: 2012 Oct 22
Publication date: 2012
Volume: 15
Issue: Suppl 3
Electronic Location ID: 18443-17
Copyright: © 2012 K. Regan et al.
Copyright year: 2012
License: This is an open-access article distributed under the terms of the Creative Commons Attribution License, which permits unrestricted use, distribution, and reproduction in any medium, provided the original work is properly cited.
License URL: http://creativecommons.org/licenses/by/2.0/

JOURNAL INFORMATION
==============================
NLM Title Abbreviation: J Int AIDS Soc
Journal ID: J Int AIDS Soc
NLM Journal ID: 101478566
Journal ID: JIAS

Journal of the International AIDS Society

EISSN: 1758-2652
Publisher: Wiley

ARTICLE INFORMATION
==============================
Article ID: 18443-18
Subjects: E21 - Using mobile phones/other methods to track patient and for programme data

WEAE0304: Mobile telemedicine for improved community-level clinical decision making, referrals and medical information transmission and storage: a pilot study in Nairobi, Kenya

Cohn J. 1 §
Xiong K. 2
1 University of Pennsylvania School of Medicine, Department of Medicine, Philadelphia, United States
2 Moi University, Nairobi, Kenya
§ Presenting author email: jecohn6@gmail.com
Electronic publication date: 2012 Oct 22
Publication date: 2012
Volume: 15
Issue: Suppl 3
Electronic Location ID: 18443-18
Copyright: © 2012 J. Cohn et al.
Copyright year: 2012
License: This is an open-access article distributed under the terms of the Creative Commons Attribution License, which permits unrestricted use, distribution, and reproduction in any medium, provided the original work is properly cited.
License URL: http://creativecommons.org/licenses/by/2.0/

JOURNAL INFORMATION
==============================
NLM Title Abbreviation: J Int AIDS Soc
Journal ID: J Int AIDS Soc
NLM Journal ID: 101478566
Journal ID: JIAS

Journal of the International AIDS Society

EISSN: 1758-2652
Publisher: Wiley

ARTICLE INFORMATION
==============================
Article ID: 18443-19
Subjects: E21 - Using mobile phones/other methods to track patient and for programme data

FRAE0104: An automated platform for delivery of CD4 results to SMS-enabled antenatal clinic printers, Botswana

Veres A. 1
Dryden-Peterson S. 2 §
van Widenfelt E. 3
Motshegwa P. 3
Mine M. 4
Moyo S. 3
Keapoletswe K. 4
Avalos A. 4
Mmalane M. 3
Shapiro R. 5
Lockman S. 2
1 Harvard University, Faculty of Arts and Sciences, Cambridge, United States
2 Brigham and Women's Hospital, Division of Infectious Diseases, Boston, United States
3 Botswana Harvard AIDS Institute, Gaborone, Botswana
4 Botswana Ministry of Health, Department of HIV/AIDS Prevention and Care, Gaborone, Botswana
5 Beth Israel Deaconess Medical Center, Boston, United States
§ Presenting author email: scott_peterson@post.harvard.edu
Electronic publication date: 2012 Oct 22
Publication date: 2012
Volume: 15
Issue: Suppl 3
Electronic Location ID: 18443-19
Copyright: © 2012 S. Dryden-Peterson et al.
Copyright year: 2012
License: This is an open-access article distributed under the terms of the Creative Commons Attribution License, which permits unrestricted use, distribution, and reproduction in any medium, provided the original work is properly cited.
License URL: http://creativecommons.org/licenses/by/2.0/

JOURNAL INFORMATION
==============================
NLM Title Abbreviation: J Int AIDS Soc
Journal ID: J Int AIDS Soc
NLM Journal ID: 101478566
Journal ID: JIAS

Journal of the International AIDS Society

EISSN: 1758-2652
Publisher: Wiley

ARTICLE INFORMATION
==============================
Article ID: 18443-20
Subjects: E21 - Using mobile phones/other methods to track patient and for programme data

TULBE02: Implementation of a wireless GPRS-based monitoring system for point-of-care CD4 testing at rural primary health facilities in Mozambique

Tobaiwa O. 1
Bollinger T. 1
Sitoe N. 2
Lehe J. 1
Peter T. 1
Jani I. 2
Quevedo J. 1 §
1 Clinton Health Access Initiative, Maputo, Mozambique
2 Instituto Nacional de Saúde, Maputo, Mozambique
§ Presenting author email: jquevedo@clintonhealthaccess.org
Electronic publication date: 2012 Oct 22
Publication date: 2012
Volume: 15
Issue: Suppl 3
Electronic Location ID: 18443-20
Copyright: © 2012 J. Quevedo et al.
Copyright year: 2012
License: This is an open-access article distributed under the terms of the Creative Commons Attribution License, which permits unrestricted use, distribution, and reproduction in any medium, provided the original work is properly cited.
License URL: http://creativecommons.org/licenses/by/2.0/

JOURNAL INFORMATION
==============================
NLM Title Abbreviation: J Int AIDS Soc
Journal ID: J Int AIDS Soc
NLM Journal ID: 101478566
Journal ID: JIAS

Journal of the International AIDS Society

EISSN: 1758-2652
Publisher: Wiley

ARTICLE INFORMATION
==============================
Article ID: 18443-21
Subjects: E25 - Strategies to increase HIV testing, and to promote linkage and retention in care

WEAE0202: A laboratory-based approach to reduce loss to follow-up of HIV-positive clients

Nwuba C.O. 1
Dagunduro T. 1 §
Umenyi C. 1
Peters B. 2
Afolayan F. 3
Abolarin O. 4
1 Management Sciences for Health, Prevention Organizational Systems, AIDS, Care and Treatment, Ilorin, Nigeria
2 Children Specialist Hospital, Laboratory Unit, Ilorin, Nigeria
3 General Hospital, Laboratory Unit, Omuaran, Nigeria
4 Specialist Hospital, Laboratory Unit, Offa, Nigeria
§ Presenting author email: cnwuba@msh.org
Electronic publication date: 2012 Oct 22
Publication date: 2012
Volume: 15
Issue: Suppl 3
Electronic Location ID: 18443-21
Copyright: © 2012 T. Dagunduro et al.
Copyright year: 2012
License: This is an open-access article distributed under the terms of the Creative Commons Attribution License, which permits unrestricted use, distribution, and reproduction in any medium, provided the original work is properly cited.
License URL: http://creativecommons.org/licenses/by/2.0/

JOURNAL INFORMATION
==============================
NLM Title Abbreviation: J Int AIDS Soc
Journal ID: J Int AIDS Soc
NLM Journal ID: 101478566
Journal ID: JIAS

Journal of the International AIDS Society

EISSN: 1758-2652
Publisher: Wiley

ARTICLE INFORMATION
==============================
Article ID: 18443-22
Subjects: E25 - Strategies to increase HIV testing, and to promote linkage and retention in care

WEAE0301: The distribution and use of cell phones to mothers of HIV-positive infants identified by the Haiti National Early Infant Diagnosis of HIV program (EID): a model for increasing adherence?

Segaren N. 1
Lewis T. 1
Desinor O. 2
Simeon E. 1
Segaren N. 3
1 Caris Foundation, Port au Prince, Haiti
2 USAID, PAP, Haiti
3 Chelsea & Westminster Hospital, London, United Kingdom
Electronic publication date: 2012 Oct 22
Publication date: 2012
Volume: 15
Issue: Suppl 3
Electronic Location ID: 18443-22
Copyright: © 2012 N. Segaren et al.
Copyright year: 2012
License: This is an open-access article distributed under the terms of the Creative Commons Attribution License, which permits unrestricted use, distribution, and reproduction in any medium, provided the original work is properly cited.
License URL: http://creativecommons.org/licenses/by/2.0/

JOURNAL INFORMATION
==============================
NLM Title Abbreviation: J Int AIDS Soc
Journal ID: J Int AIDS Soc
NLM Journal ID: 101478566
Journal ID: JIAS

Journal of the International AIDS Society

EISSN: 1758-2652
Publisher: Wiley

ARTICLE INFORMATION
==============================
Article ID: 18443-23
Subjects: E25 - Strategies to increase HIV testing, and to promote linkage and retention in care

MOPDE0203: Risk factors for late-stage HIV disease presentation at initial HIV diagnosis in Durban, South Africa

Drain P. 1 §
Losina E. 2
Chetty S. 3
Giddy J. 3
Ross D. 4
Katz J. 5
Coleman S. 6
Bogart L. 7
Freedberg K. 2
Walensky R. 2
Ingrid B. 1
1 Massachusetts General Hospital, Boston, United States
2 Harvard Center for AIDS Research, Boston, United States
3 McCord Hospital, Durban, South Africa
4 St. Mary's Hospital, Durban, South Africa
5 Brigham & Women's Hospital, Boston, United States
6 Boston University School of Public Health, Boston, United States
7 Children's Hospital Boston, Boston, United States
§ Presenting author email: pkdrain@gmail.com
Electronic publication date: 2012 Oct 22
Publication date: 2012
Volume: 15
Issue: Suppl 3
Electronic Location ID: 18443-23
Copyright: © 2012 P. Drain et al.
Copyright year: 2012
License: This is an open-access article distributed under the terms of the Creative Commons Attribution License, which permits unrestricted use, distribution, and reproduction in any medium, provided the original work is properly cited.
License URL: http://creativecommons.org/licenses/by/2.0/

JOURNAL INFORMATION
==============================
NLM Title Abbreviation: J Int AIDS Soc
Journal ID: J Int AIDS Soc
NLM Journal ID: 101478566
Journal ID: JIAS

Journal of the International AIDS Society

EISSN: 1758-2652
Publisher: Wiley

ARTICLE INFORMATION
==============================
Article ID: 18443-24
Subjects: E25 - Strategies to increase HIV testing, and to promote linkage and retention in care

THPDE0301: Towards universal awareness of HIV status: a systematic review on uptake of home-based HIV testing in sub-Saharan Africa

Sabapathy K. 1 2 §
Van den Bergh R. 3
Fidler S. 2
Hayes R. 1
Ford N. 4 5
1 London School of Hygiene and Tropical Medicine, London, United Kingdom
2 Imperial College, London, United Kingdom
3 Medecins Sans Frontieres, Brussels, Belgium
4 Medecins Sans Frontieres, London, United Kingdom
5 University of Cape Town, Cape Town, South Africa
§ Presenting author email: kalpana.sabapathy@gmail.com
Electronic publication date: 2012 Oct 22
Publication date: 2012
Volume: 15
Issue: Suppl 3
Electronic Location ID: 18443-24
Copyright: © 2012 K. Sabapathy et al.
Copyright year: 2012
License: This is an open-access article distributed under the terms of the Creative Commons Attribution License, which permits unrestricted use, distribution, and reproduction in any medium, provided the original work is properly cited.
License URL: http://creativecommons.org/licenses/by/2.0/

JOURNAL INFORMATION
==============================
NLM Title Abbreviation: J Int AIDS Soc
Journal ID: J Int AIDS Soc
NLM Journal ID: 101478566
Journal ID: JIAS

Journal of the International AIDS Society

EISSN: 1758-2652
Publisher: Wiley

ARTICLE INFORMATION
==============================
Article ID: 18443-25
Subjects: E25 - Strategies to increase HIV testing, and to promote linkage and retention in care

THPDE0302: Thinking outside the docs: expanding access to HIV testing services through the delivery of HIV testing at the Department of Motor Vehicles in Washington, D.C.

Fulwood Wood A. 1 §
Parris D. 1
Terrell Hamilton F. 1
Brockington S. 1
1 Family & Medical Counseling Service, Inc., Washington, United States
§ Presenting author email: afulwood@fmcsinc.org
Electronic publication date: 2012 Oct 22
Publication date: 2012
Volume: 15
Issue: Suppl 3
Electronic Location ID: 18443-25
Copyright: © 2012 A. Fulwood Wood et al.
Copyright year: 2012
License: This is an open-access article distributed under the terms of the Creative Commons Attribution License, which permits unrestricted use, distribution, and reproduction in any medium, provided the original work is properly cited.
License URL: http://creativecommons.org/licenses/by/2.0/

JOURNAL INFORMATION
==============================
NLM Title Abbreviation: J Int AIDS Soc
Journal ID: J Int AIDS Soc
NLM Journal ID: 101478566
Journal ID: JIAS

Journal of the International AIDS Society

EISSN: 1758-2652
Publisher: Wiley

ARTICLE INFORMATION
==============================
Article ID: 18443-26
Subjects: E25 - Strategies to increase HIV testing, and to promote linkage and retention in care

THPDE0304: The national HIV counselling and testing campaign and treatment expansion in South Africa: a return on investments in combination prevention

Mbengashe T. 1
Nevhutalu Z. 2
Chipimo M. 3
Chidarikire T. 1
Diseko L. 1
1 National Department of Health, HIV Cluster, Pretoria, South Africa
2 South Africa National AIDS Council, Pretoria, South Africa
3 UNAIDS, Pretoria Country Office, Pretoria, South Africa
Electronic publication date: 2012 Oct 22
Publication date: 2012
Volume: 15
Issue: Suppl 3
Electronic Location ID: 18443-26
Copyright: © 2012 T. Mbengashe et al.
Copyright year: 2012
License: This is an open-access article distributed under the terms of the Creative Commons Attribution License, which permits unrestricted use, distribution, and reproduction in any medium, provided the original work is properly cited.
License URL: http://creativecommons.org/licenses/by/2.0/

JOURNAL INFORMATION
==============================
NLM Title Abbreviation: J Int AIDS Soc
Journal ID: J Int AIDS Soc
NLM Journal ID: 101478566
Journal ID: JIAS

Journal of the International AIDS Society

EISSN: 1758-2652
Publisher: Wiley

ARTICLE INFORMATION
==============================
Article ID: 18443-27
Subjects: E25 - Strategies to increase HIV testing, and to promote linkage and retention in care

THPDE0306: Announcing a new method of HIV testing: the planning of robust home testing for HIV combined with internet counseling

van der Helm J. 1 §
Zuure F. 1
Fennema H. 2
Davidovich U. 1
Prins M. 1 3
on behalf of the HIVTest@Home study group
1 Health Service Amsterdam, HIV/STI Research, Amsterdam, Netherlands
2 Health Service Amsterdam, Amsterdam, Netherlands
3 Academic Medical Center, University of Amsterdam, Amsterdam, Netherlands
§ Presenting author email: jvdhelm@ggd.amsterdam.nl
Electronic publication date: 2012 Oct 22
Publication date: 2012
Volume: 15
Issue: Suppl 3
Electronic Location ID: 18443-27
Copyright: © 2012 J. van der Helm et al.
Copyright year: 2012
License: This is an open-access article distributed under the terms of the Creative Commons Attribution License, which permits unrestricted use, distribution, and reproduction in any medium, provided the original work is properly cited.
License URL: http://creativecommons.org/licenses/by/2.0/

JOURNAL INFORMATION
==============================
NLM Title Abbreviation: J Int AIDS Soc
Journal ID: J Int AIDS Soc
NLM Journal ID: 101478566
Journal ID: JIAS

Journal of the International AIDS Society

EISSN: 1758-2652
Publisher: Wiley

ARTICLE INFORMATION
==============================
Article ID: 18443-28
Subjects: E26 - Co-designing and implementing programmes at national and regional scale

TULBE03: HIV treatment as prevention: driving health system development and improved health outcomes in British Columbia

Panessa C. 1 §
O'Briain W. 2
McGowan G. 2
Montaner J. 3
Kendall P. 4
1 British Columbia Ministry of Health, Population and Public Health, Vancouver, Canada
2 British Columbia Ministry of Health, Population and Public Health, Victoria, Canada
3 BC Centre for Excellence in HIV/AIDS, Vancouver, Canada
4 Office of the Provincial Health Officer, Ministry of Health, Victoria, Canada
§ Presenting author email: ciro.panessa@gov.bc.ca
Electronic publication date: 2012 Oct 22
Publication date: 2012
Volume: 15
Issue: Suppl 3
Electronic Location ID: 18443-28
Copyright: © 2012 C. Panessa et al.
Copyright year: 2012
License: This is an open-access article distributed under the terms of the Creative Commons Attribution License, which permits unrestricted use, distribution, and reproduction in any medium, provided the original work is properly cited.
License URL: http://creativecommons.org/licenses/by/2.0/

JOURNAL INFORMATION
==============================
NLM Title Abbreviation: J Int AIDS Soc
Journal ID: J Int AIDS Soc
NLM Journal ID: 101478566
Journal ID: JIAS

Journal of the International AIDS Society

EISSN: 1758-2652
Publisher: Wiley

ARTICLE INFORMATION
==============================
Article ID: 18443-29
Subjects: E26 - Co-designing and implementing programmes at national and regional scale

WEPDE0105: 'Less is more’: assessing the effectiveness of a HIV prevention program for long distance trucker drivers in India after a re-designing in implementation structure

Tirumalasetti V.R. 1 §
Mahapatra B. 2
Juneja S. 1
Singh I. 1
1 TCI Foundation, HIV/AIDS Communicatios, Delhi, India
2 Population Council, New Delhi, India
§ Presenting author email: vasudha.tcif@tcil.com
Electronic publication date: 2012 Oct 22
Publication date: 2012
Volume: 15
Issue: Suppl 3
Electronic Location ID: 18443-29
Copyright: © 2012 V. R. Tirumalasetti et al.
Copyright year: 2012
License: This is an open-access article distributed under the terms of the Creative Commons Attribution License, which permits unrestricted use, distribution, and reproduction in any medium, provided the original work is properly cited.
License URL: http://creativecommons.org/licenses/by/2.0/

JOURNAL INFORMATION
==============================
NLM Title Abbreviation: J Int AIDS Soc
Journal ID: J Int AIDS Soc
NLM Journal ID: 101478566
Journal ID: JIAS

Journal of the International AIDS Society

EISSN: 1758-2652
Publisher: Wiley

ARTICLE INFORMATION
==============================
Article ID: 18443-30
Subjects: E27 - Capacitating public health systems to deliver HIV care at scale

TULBE04: Changes in population-level HIV RNA distribution one year after implementation of key components of an HIV ′test and treat′ strategy in rural Uganda

Jain V. 1 2 §
Kwarisiima D. 2 3
Liegler T. 1
Clark T. 1 2
Chamie G. 1 2
Kabami J. 2
Black D. 1 2
Amanyire G. 2 3
Byonanebye D. 2 3
Geng E. 1 2
Thirumurthy H. 2 4
Petersen M. 2 5
Charlebois E. 2 6
Kamya M. 2 7
Havlir D. 1 2
and the SEARCH Collaboration
1 University of California San Francisco, HIV/AIDS Division, San Francisco General Hospital, San Francisco, United States
2 Makerere University-UCSF Research Collaboration, Kampala, Uganda
3 Mulago-Mbarara Joint AIDS Program, Mbarara, Uganda
4 Gillings School of Global Public Health, University of North Carolina, Chapel Hill, United States
5 School of Public Health, University of California, Berkeley, Division of Biostatistics, Berkeley, United States
6 Center for AIDS Prevention Studies (CAPS), University of California San Francisco, San Francisco, United States
7 Makerere University School of Medicine, Kampala, Uganda
§ Presenting author email: vivek.jain@ucsf.edu
Electronic publication date: 2012 Oct 22
Publication date: 2012
Volume: 15
Issue: Suppl 3
Electronic Location ID: 18443-30
Copyright: © 2012 V. Jain et al.
Copyright year: 2012
License: This is an open-access article distributed under the terms of the Creative Commons Attribution License, which permits unrestricted use, distribution, and reproduction in any medium, provided the original work is properly cited.
License URL: http://creativecommons.org/licenses/by/2.0/

JOURNAL INFORMATION
==============================
NLM Title Abbreviation: J Int AIDS Soc
Journal ID: J Int AIDS Soc
NLM Journal ID: 101478566
Journal ID: JIAS

Journal of the International AIDS Society

EISSN: 1758-2652
Publisher: Wiley

ARTICLE INFORMATION
==============================
Article ID: 18443-31
Subjects: E29 - International funding for HIV scale-up: following the money

MOAE0306: Funding universal access to antiretroviral treatment through a ‘Global Health Charge’ on alcohol and tobacco consumption: feasibility in the 20 countries with the largest HIV epidemics

Hill A. 1 §
Sawyer W. 2
1 Liverpool University, Pharmacology and Therapeutics, London, United Kingdom
2 MetaVirology Ltd, Statistics, London, United Kingdom
§ Presenting author email: microhaart@aol.com
Electronic publication date: 2012 Oct 22
Publication date: 2012
Volume: 15
Issue: Suppl 3
Electronic Location ID: 18443-31
Copyright: © 2012 A. Hill et al.
Copyright year: 2012
License: This is an open-access article distributed under the terms of the Creative Commons Attribution License, which permits unrestricted use, distribution, and reproduction in any medium, provided the original work is properly cited.
License URL: http://creativecommons.org/licenses/by/2.0/

JOURNAL INFORMATION
==============================
NLM Title Abbreviation: J Int AIDS Soc
Journal ID: J Int AIDS Soc
NLM Journal ID: 101478566
Journal ID: JIAS

Journal of the International AIDS Society

EISSN: 1758-2652
Publisher: Wiley

ARTICLE INFORMATION
==============================
Article ID: 18443-32
Subjects: E31 - Impact of health insurance schemes, co-payments and out-of-pocket payments on access, adherence and outcomes

MOPDE0201: Time and money: the costs of utilizing HIV and TB treatment and care in rural KwaZulu-Natal

Chimbindi N.Z. 1 2 §
Newell M.-L. 1 3
Bärnighausen T. 1 4
1 Africa Centre for Health and Population Studies, Research/Scientific, Mtubatuba, South Africa
2 University of Witwatersrand, School of Public Health, Johannesburg, South Africa
3 University College London, Institute of Child Health, London, United Kingdom
4 Harvard School of Public Health, Global Health and Population, Boston, United States
§ Presenting author email: nchimbindi@africacentre.ac.za
Electronic publication date: 2012 Oct 22
Publication date: 2012
Volume: 15
Issue: Suppl 3
Electronic Location ID: 18443-32
Copyright: © 2012 N. Z. Chimbindi et al.
Copyright year: 2012
License: This is an open-access article distributed under the terms of the Creative Commons Attribution License, which permits unrestricted use, distribution, and reproduction in any medium, provided the original work is properly cited.
License URL: http://creativecommons.org/licenses/by/2.0/

JOURNAL INFORMATION
==============================
NLM Title Abbreviation: J Int AIDS Soc
Journal ID: J Int AIDS Soc
NLM Journal ID: 101478566
Journal ID: JIAS

Journal of the International AIDS Society

EISSN: 1758-2652
Publisher: Wiley

ARTICLE INFORMATION
==============================
Article ID: 18443-33
Subjects: E33 - Social protection

MOPDE0205: Economic spillover effects of ART on rural South African households

Bor J. 1 2 §
Bärnighausen T. 1 2
Tanser F. 2
Newell M.-L. 3
1 Harvard School of Public Health, Global Health and Population, Boston, United States
2 Africa Centre for Health and Population Studies, University of KwaZulu-Natal, Mtubatuba, South Africa
3 Centre for Paediatric Epidemiology and Biostatistics, University College of London Institute of Child Health, London, United Kingdom
§ Presenting author email: jbor@hsph.harvard.edu
Electronic publication date: 2012 Oct 22
Publication date: 2012
Volume: 15
Issue: Suppl 3
Electronic Location ID: 18443-33
Copyright: © 2012 J. Bor et al.
Copyright year: 2012
License: This is an open-access article distributed under the terms of the Creative Commons Attribution License, which permits unrestricted use, distribution, and reproduction in any medium, provided the original work is properly cited.
License URL: http://creativecommons.org/licenses/by/2.0/

JOURNAL INFORMATION
==============================
NLM Title Abbreviation: J Int AIDS Soc
Journal ID: J Int AIDS Soc
NLM Journal ID: 101478566
Journal ID: JIAS

Journal of the International AIDS Society

EISSN: 1758-2652
Publisher: Wiley

ARTICLE INFORMATION
==============================
Article ID: 18443-34
Subjects: E34 - Cost-effectiveness of optimizing diagnostics and monitoring tools

TUAE0101: Cost-effectiveness of lateral-flow urine LAM for TB diagnosis in HIV-positive South African adults

Sun D. 1 §
Dorman S. 2 3
Shah M. 2 3
Manabe Y. 3 4
Dowdy D. 1 3
1 Johns Hopkins Bloomberg School of Public Health, Epidemiology, Baltimore, United States
2 Johns Hopkins School of Medicine, Medicine, Baltimore, United States
3 Center for Tuberculosis Research, Baltimore, United States
4 Infectious Diseases Institute, Kampala, Uganda
§ Presenting author email: sandydisun@gmail.com
Electronic publication date: 2012 Oct 22
Publication date: 2012
Volume: 15
Issue: Suppl 3
Electronic Location ID: 18443-34
Copyright: © 2012 D. Sun et al.
Copyright year: 2012
License: This is an open-access article distributed under the terms of the Creative Commons Attribution License, which permits unrestricted use, distribution, and reproduction in any medium, provided the original work is properly cited.
License URL: http://creativecommons.org/licenses/by/2.0/

JOURNAL INFORMATION
==============================
NLM Title Abbreviation: J Int AIDS Soc
Journal ID: J Int AIDS Soc
NLM Journal ID: 101478566
Journal ID: JIAS

Journal of the International AIDS Society

EISSN: 1758-2652
Publisher: Wiley

ARTICLE INFORMATION
==============================
Article ID: 18443-35
Subjects: E34 - Cost-effectiveness of optimizing diagnostics and monitoring tools

TUAE0105: Routine HIV screening in Portugal: clinical impact and cost-effectiveness

Yazdanpanah Y. 1 §
Perelman J. 2
Alves J. 2
Mansinho K. 3
Dilorenzo M.A. 4
Park J.-E. 4
Losina E. 5 6 7
Walensky R.P. 4 5
Noubary F. 4
Barros H. 8
Freedberg K.A. 4 6 7
Paltiel A.D. 9
1 Hospital Bichat Claude Bernard, Paris, France
2 National School of Public Health, Nova University of Lisbon, Lisbon, Portugal
3 Hospital Center Lisboa Ocidental, Lisbon, Portugal
4 Massachusetts General Hospital, Boston, United States
5 Brigham and Women's Hospital, Boston, United States
6 Harvard Medical School, Boston, United States
7 Boston University School of Public Health, Boston, United States
8 Institute of Public Health, Porto University, Porto, Portugal
9 Yale University, New Haven, United States
§ Presenting author email: yazdan.yazdanpanah@bch.aphp.fr
Electronic publication date: 2012 Oct 22
Publication date: 2012
Volume: 15
Issue: Suppl 3
Electronic Location ID: 18443-35
Copyright: © 2012 Y. Yazdanpanah et al.
Copyright year: 2012
License: This is an open-access article distributed under the terms of the Creative Commons Attribution License, which permits unrestricted use, distribution, and reproduction in any medium, provided the original work is properly cited.
License URL: http://creativecommons.org/licenses/by/2.0/

JOURNAL INFORMATION
==============================
NLM Title Abbreviation: J Int AIDS Soc
Journal ID: J Int AIDS Soc
NLM Journal ID: 101478566
Journal ID: JIAS

Journal of the International AIDS Society

EISSN: 1758-2652
Publisher: Wiley

ARTICLE INFORMATION
==============================
Article ID: 18443-36
Subjects: E34 - Cost-effectiveness of optimizing diagnostics and monitoring tools

TUPDE0201: Cost effectiveness evaluation of alternative monitoring strategies for antiretroviral therapy in low-resource settings: monitoring of HIV viral loads, CD4 cell counts and clinical assessments versus clinical monitoring alone (STRATALL ANRS 12110/ESTHER trial in Cameroon)

Boyer S. 1 2 3
March L. 1 2 3
Kouanfack C. 4
Laborde-Balen G. 5
Marino P. 1 2
Aghokeng A. 6 7
Mpoudi-Ngole E. 7
Koulla-Shiro S. 4 8
Delaporte E. 6 9
Carrieri M.P. 1 2 3
Spire B. 1 2 3
Laurent C. 6
Moatti J.-P. 1 2 3 §
Stratall ANRS 12110/ESTHER Study Group
1 INSERM, U912 (SESSTIM), Marseille, France
2 Aix Marseille Universit, IRD, UMR-S912, Marseille, France
3 ORS PACA, Observatoire Rgional de la Sant Provence Alpes Cte d'Azur, Marseille, France
4 Central Hospital, UMI 233, Yaound, Cameroon
5 UMI 233 Yaound, Cameroon, French Ministry of Foreign Affairs, Yaound, Cameroon
6 Institut de Recherche pour le Dveloppement (IRD), University Montpellier 1, UMI 233, Montpellier, France
7 Virology Laboratory IRD/IMPM/CREMER, Yaound, Cameroon
8 University Yaound 1, Yaound, Cameroon
9 Department of Infectious and Tropical Diseases, University Hospital, Montpellier, France
§ Presenting author email: jean-paul.moatti@inserm.fr
Electronic publication date: 2012 Oct 22
Publication date: 2012
Volume: 15
Issue: Suppl 3
Electronic Location ID: 18443-36
Copyright: © 2012 J.-P. Moatti et al.
Copyright year: 2012
License: This is an open-access article distributed under the terms of the Creative Commons Attribution License, which permits unrestricted use, distribution, and reproduction in any medium, provided the original work is properly cited.
License URL: http://creativecommons.org/licenses/by/2.0/

JOURNAL INFORMATION
==============================
NLM Title Abbreviation: J Int AIDS Soc
Journal ID: J Int AIDS Soc
NLM Journal ID: 101478566
Journal ID: JIAS

Journal of the International AIDS Society

EISSN: 1758-2652
Publisher: Wiley

ARTICLE INFORMATION
==============================
Article ID: 18443-37
Subjects: E36 - Cost-effectiveness of management of health care systems

TUAE0102: Cost-effectiveness of strategies to promote identification of HIV cases: economic analysis of a controlled trial in China

Zeng G. 1 §
Lu F. 2
Khoshnood K. 3
Wu Z. 2
1 Chinese Center for Disease Control and Prevention, Beijing, China
2 National Center for AIDS/STD Control and Prevention, Chinese Center for Disease Control and Prevention, Beijing, China
3 Yale University, New Haven, United States
§ Presenting author email: hxydzg@163.com
Electronic publication date: 2012 Oct 22
Publication date: 2012
Volume: 15
Issue: Suppl 3
Electronic Location ID: 18443-37
Copyright: © 2012 G. Zeng et al.
Copyright year: 2012
License: This is an open-access article distributed under the terms of the Creative Commons Attribution License, which permits unrestricted use, distribution, and reproduction in any medium, provided the original work is properly cited.
License URL: http://creativecommons.org/licenses/by/2.0/

JOURNAL INFORMATION
==============================
NLM Title Abbreviation: J Int AIDS Soc
Journal ID: J Int AIDS Soc
NLM Journal ID: 101478566
Journal ID: JIAS

Journal of the International AIDS Society

EISSN: 1758-2652
Publisher: Wiley

ARTICLE INFORMATION
==============================
Article ID: 18443-38
Subjects: E37 - Cost-effectiveness of programmes building individual, community and institutional capacity

TUAE0103: Cost-effectiveness of test and treat prevention in a high HIV prevalence U.S. city

Escaleira F. 1 2
Birger R. 3
Hallett T. 4
Grenfell B. 3
Hodder S. 1
Sinha A. 5
1 New Jersey Medical School, UMDNJ, Medicine, Division of Infectious Diseases, Newark, United States
2 UMDNJ School of Public Health, Piscataway, United States
3 Princeton University, Ecology and Evolutionary Biology, Princeton, United States
4 Department of Infectious Disease Epidemiology, Imperial College London, London, United Kingdom
5 New Jersey Medical School, UMDNJ, Preventive Medicine and Community Health, Newark, United States
Electronic publication date: 2012 Oct 22
Publication date: 2012
Volume: 15
Issue: Suppl 3
Electronic Location ID: 18443-38
Copyright: © 2012 F. Escaleira et al.
Copyright year: 2012
License: This is an open-access article distributed under the terms of the Creative Commons Attribution License, which permits unrestricted use, distribution, and reproduction in any medium, provided the original work is properly cited.
License URL: http://creativecommons.org/licenses/by/2.0/

JOURNAL INFORMATION
==============================
NLM Title Abbreviation: J Int AIDS Soc
Journal ID: J Int AIDS Soc
NLM Journal ID: 101478566
Journal ID: JIAS

Journal of the International AIDS Society

EISSN: 1758-2652
Publisher: Wiley

ARTICLE INFORMATION
==============================
Article ID: 18443-39
Subjects: E37 - Cost-effectiveness of programmes building individual, community and institutional capacity

TUPDE0103: Value for money of structural interventions: going beyond HIV-only cost-effectiveness analysis

Remme M. 1 §
Vassall A. 1
Lutz B. 2
Watts C. 1
1 London School of Hygiene & Tropical Medicine, Global Health & Development, London, United Kingdom
2 UNDP, New York, United States
§ Presenting author email: michelle.remme@lshtm.ac.uk
Electronic publication date: 2012 Oct 22
Publication date: 2012
Volume: 15
Issue: Suppl 3
Electronic Location ID: 18443-39
Copyright: © 2012 M. Remme et al.
Copyright year: 2012
License: This is an open-access article distributed under the terms of the Creative Commons Attribution License, which permits unrestricted use, distribution, and reproduction in any medium, provided the original work is properly cited.
License URL: http://creativecommons.org/licenses/by/2.0/

JOURNAL INFORMATION
==============================
NLM Title Abbreviation: J Int AIDS Soc
Journal ID: J Int AIDS Soc
NLM Journal ID: 101478566
Journal ID: JIAS

Journal of the International AIDS Society

EISSN: 1758-2652
Publisher: Wiley

ARTICLE INFORMATION
==============================
Article ID: 18443-40
Subjects: E38 - Effects of adult and paediatric HIV treatment on earned and family income

MOPDE0204: Impact of antiretroviral therapy (ART) on socio-economic status and productivity of HIV-positive individuals/households in a private setting in India

Visnegarwala F. 1 2 §
Nalli A. 2
Babu R. 2
Satish S. 2
Pradeep L. 2
Alexander G. 2
1 Indian Institute of Health Management and Research, Public Health, Bangalore, India
2 Action Service and Hope for AIDS (ASHA) Foundation, Research, Bangalore, India
§ Presenting author email: fehmidav@gmail.com
Electronic publication date: 2012 Oct 22
Publication date: 2012
Volume: 15
Issue: Suppl 3
Electronic Location ID: 18443-40
Copyright: © 2012 Y. Visnegarwala et al.
Copyright year: 2012
License: This is an open-access article distributed under the terms of the Creative Commons Attribution License, which permits unrestricted use, distribution, and reproduction in any medium, provided the original work is properly cited.
License URL: http://creativecommons.org/licenses/by/2.0/

JOURNAL INFORMATION
==============================
NLM Title Abbreviation: J Int AIDS Soc
Journal ID: J Int AIDS Soc
NLM Journal ID: 101478566
Journal ID: JIAS

Journal of the International AIDS Society

EISSN: 1758-2652
Publisher: Wiley

ARTICLE INFORMATION
==============================
Article ID: 18443-41
Subjects: E41 - Pharmacoeconomics and cost effectiveness

FRAE0102: The cost-effectiveness of maternal and infant antiretroviral regimens to prevent vertical HIV transmission in Malawi

Revill P. 1 §
Sculpher M. 1
Walker S. 1
Chasela C.S. 2
Kayira D. 2
Hosseinipour M.C. 2 3
Merry C. 4
Barry M. 4
King C.K. 5
Jamieson D.J. 5
Gibb D.M. 6
van der Horst C. 3
Ryan M. 4
1 University of York, Centre for Health Economics, York, United Kingdom
2 University of North Carolina Project, Lilongwe, Malawi
3 University of North Carolina at Chapel Hill, Division of Infectious Diseases, Centre for AIDS Research, Chapel Hill, United States
4 Trinity College Dublin, Department of Pharmacology and Therapeutics, School of Medicine, Dublin, Ireland
5 Centers for Disease Control and Prevention (CDC), Atlanta, United States
6 Medical Research Council, Clinical Trials Unit, London, United Kingdom
§ Presenting author email: paul.revill@york.ac.uk
Electronic publication date: 2012 Oct 22
Publication date: 2012
Volume: 15
Issue: Suppl 3
Electronic Location ID: 18443-41
Copyright: © 2012 P. Revill et al.
Copyright year: 2012
License: This is an open-access article distributed under the terms of the Creative Commons Attribution License, which permits unrestricted use, distribution, and reproduction in any medium, provided the original work is properly cited.
License URL: http://creativecommons.org/licenses/by/2.0/

JOURNAL INFORMATION
==============================
NLM Title Abbreviation: J Int AIDS Soc
Journal ID: J Int AIDS Soc
NLM Journal ID: 101478566
Journal ID: JIAS

Journal of the International AIDS Society

EISSN: 1758-2652
Publisher: Wiley

ARTICLE INFORMATION
==============================
Article ID: 18443-42
Subjects: E41 - Pharmacoeconomics and cost effectiveness

FRLBX06: The clinical and economic impact of a generic first-line antiretroviral regimen in the U.S.

Walensky R.P. 1 §
Sax P.E. 2
Nakamura Y.M. 3
Weinstein M.C. 4
Pei P.P. 3
Freedberg K.A. 1
Paltiel A.D. 5
Schackman B.R. 6
1 Massachusetts General Hospital, Harvard Medical School, Boston, United States
2 Brigham and Women's Hospital, Harvard Medical School, Boston, United States
3 Massachusetts General Hospital, Boston, United States
4 Harvard School of Public Health, Boston, United States
5 Yale School of Medicine, New Haven, United States
6 Weill Cornell Medical College, New York, United States
§ Presenting author email: rwalensky@partners.org
Electronic publication date: 2012 Oct 22
Publication date: 2012
Volume: 15
Issue: Suppl 3
Electronic Location ID: 18443-42
Copyright: © 2012 R.P. Walensky et al.
Copyright year: 2012
License: This is an open-access article distributed under the terms of the Creative Commons Attribution License, which permits unrestricted use, distribution, and reproduction in any medium, provided the original work is properly cited.
License URL: http://creativecommons.org/licenses/by/2.0/

JOURNAL INFORMATION
==============================
NLM Title Abbreviation: J Int AIDS Soc
Journal ID: J Int AIDS Soc
NLM Journal ID: 101478566
Journal ID: JIAS

Journal of the International AIDS Society

EISSN: 1758-2652
Publisher: Wiley

ARTICLE INFORMATION
==============================
Article ID: 18443-43
Subjects: E42 - Effects of public-private partnerships, and working with faith-based groups and traditional healers on HIV outcomes

TULBE01: Quantifying the role of private health providers in HIV testing: analysis of data from 18 countries

Johnson D. 1
Cheng X. 1
Sulzbach S. 1 §
1 Abt Associates Inc., Bethesda, United States
§ Presenting author email: sara_sulzbach@abtassoc.com
Electronic publication date: 2012 Oct 22
Publication date: 2012
Volume: 15
Issue: Suppl 3
Electronic Location ID: 18443-43
Copyright: © 2012 S. Sulzbach et al.
Copyright year: 2012
License: This is an open-access article distributed under the terms of the Creative Commons Attribution License, which permits unrestricted use, distribution, and reproduction in any medium, provided the original work is properly cited.
License URL: http://creativecommons.org/licenses/by/2.0/

JOURNAL INFORMATION
==============================
NLM Title Abbreviation: J Int AIDS Soc
Journal ID: J Int AIDS Soc
NLM Journal ID: 101478566
Journal ID: JIAS

Journal of the International AIDS Society

EISSN: 1758-2652
Publisher: Wiley

ARTICLE INFORMATION
==============================
Article ID: 18443-44
Subjects: E47 - Human resources development for prevention, treatment, care and multi-sectoral responses

THPDE0104: Helping people deliver: how introducing pre-service training in supply chain management has strengthened local capacity, produced cost-savings and improved patients’ lives

Hasselberg E. 1 §
Chavez J. 2
1 Supply Chain Management System (SCMS), Boston, United States
2 Supply Chain Management System (SCMS), Lusaka, Zambia
§ Presenting author email: ehasselberg@pfscm.org
Electronic publication date: 2012 Oct 22
Publication date: 2012
Volume: 15
Issue: Suppl 3
Electronic Location ID: 18443-44
Copyright: © 2012 E. Hasselberg et al.
Copyright year: 2012
License: This is an open-access article distributed under the terms of the Creative Commons Attribution License, which permits unrestricted use, distribution, and reproduction in any medium, provided the original work is properly cited.
License URL: http://creativecommons.org/licenses/by/2.0/

JOURNAL INFORMATION
==============================
NLM Title Abbreviation: J Int AIDS Soc
Journal ID: J Int AIDS Soc
NLM Journal ID: 101478566
Journal ID: JIAS

Journal of the International AIDS Society

EISSN: 1758-2652
Publisher: Wiley

ARTICLE INFORMATION
==============================
Article ID: 18443-45
Subjects: E48 - Community coordinating mechanisms

WEPDE0205: Reducing harassment amongst sex workers: Aastha experiences of community committees in addressing crises in Mumbai, India

Nidhi G. 1 §
Ranebennur V. 2
Gaikwad D.S.S. 3
Bhende A. 4
1 FHI 360, India, Program-Community Mobilization and Advocacy, Mumbai, India
2 FHI 360, India, Program, Mumbai, India
3 FHI 360, India, Directorate, Mumbai, India
4 FHI 360, Aastha, Mumbai, India
§ Presenting author email: gnidhi@fhiindia.org
Electronic publication date: 2012 Oct 22
Publication date: 2012
Volume: 15
Issue: Suppl 3
Electronic Location ID: 18443-45
Copyright: © 2012 G. Nidhi et al.
Copyright year: 2012
License: This is an open-access article distributed under the terms of the Creative Commons Attribution License, which permits unrestricted use, distribution, and reproduction in any medium, provided the original work is properly cited.
License URL: http://creativecommons.org/licenses/by/2.0/

JOURNAL INFORMATION
==============================
NLM Title Abbreviation: J Int AIDS Soc
Journal ID: J Int AIDS Soc
NLM Journal ID: 101478566
Journal ID: JIAS

Journal of the International AIDS Society

EISSN: 1758-2652
Publisher: Wiley

ARTICLE INFORMATION
==============================
Article ID: 18443-46
Subjects: E49 - Peer programmes and organizations

THPDE0305: Inmate peer educators are essential to prison-based HIV testing and TB screening in Zambia

Maggard K. 1 2
Hatwiinda S. 1
Phiri W. 1
Morse J. 1 2
Turnbull E. 1 3
Topp S. 1 4
Roberts S. 1 2
Samungole G. 5
Kapata N. 5
Chileshe C. 6
Harris J. 1 7
Reid S. 1 2
Henostroza G. 1 2 §
1 Centre for Infectious Disease Research in Zambia, Lusaka, Zambia
2 University of Alabama at Birmingham School of Medicine, Birmingham, United States
3 Barts and the London School of Medicine and Dentistry, Queen Mary, University of London, London, United Kingdom
4 Nossal Institute for Global Health, University of Melbourne, Melbourne, Australia
5 Ministry of Health, Lusaka, Zambia
6 Zambia Prisons Service, Kabwe, Zambia
7 University of Alabama at Birmingham School of Public Health, Birmingham, United States
§ Presenting author email: germanh@uab.edu
Electronic publication date: 2012 Oct 22
Publication date: 2012
Volume: 15
Issue: Suppl 3
Electronic Location ID: 18443-46
Copyright: © 2012 G. Henostroza et al.
Copyright year: 2012
License: This is an open-access article distributed under the terms of the Creative Commons Attribution License, which permits unrestricted use, distribution, and reproduction in any medium, provided the original work is properly cited.
License URL: http://creativecommons.org/licenses/by/2.0/

JOURNAL INFORMATION
==============================
NLM Title Abbreviation: J Int AIDS Soc
Journal ID: J Int AIDS Soc
NLM Journal ID: 101478566
Journal ID: JIAS

Journal of the International AIDS Society

EISSN: 1758-2652
Publisher: Wiley

ARTICLE INFORMATION
==============================
Article ID: 18443-47
Subjects: E53 - Decentralization of services

WEAE0205: Outcomes of antiretroviral treatment programs in rural Lesotho: health centers and hospitals compared

Labhardt N.D. 1
Sello M. 2
Mohlaba M. 3
Keiser O. 4
Pfeiffer K. 5
Egger M. 4
Ehmer J. 5
Wandeler G. 4 6 §
1 SolidarMed, Seboche Hospital, Botha-Bothe, Lesotho
2 SolidarMed, Thaba-Tseka, Lesotho
3 Seboche Hospital, Primary Health Care Department, Botha-Bothe, Lesotho
4 University of Bern, Institute of Social and Preventive Medicine (ISPM), Bern, Switzerland
5 SolidarMed, Lucerne, Switzerland
6 University Hospital and University of Bern, Clinic for Infectious Diseases, Bern, Switzerland
§ Presenting author email: gwandeler@ispm.unibe.ch
Electronic publication date: 2012 Oct 22
Publication date: 2012
Volume: 15
Issue: Suppl 3
Electronic Location ID: 18443-47
Copyright: © 2012 G. Wandeler et al.
Copyright year: 2012
License: This is an open-access article distributed under the terms of the Creative Commons Attribution License, which permits unrestricted use, distribution, and reproduction in any medium, provided the original work is properly cited.
License URL: http://creativecommons.org/licenses/by/2.0/

JOURNAL INFORMATION
==============================
NLM Title Abbreviation: J Int AIDS Soc
Journal ID: J Int AIDS Soc
NLM Journal ID: 101478566
Journal ID: JIAS

Journal of the International AIDS Society

EISSN: 1758-2652
Publisher: Wiley

ARTICLE INFORMATION
==============================
Article ID: 18443-48
Subjects: E53 - Decentralization of services

WEAE0206: High rates of loss to follow-up during first year of pre-antiretroviral therapy for HIV at primary health care level in rural Uganda

Scheibe F.J.B. 1 2
Muhamadi L. 3 4 5
Waiswa P. 4 5 6
Mueller O. 1
Neuhann H.W.F. 1 §
1 University of Heidelberg, Institute of Public Health, Heidelberg, Germany
2 University of Heidelberg, Surgical Clinic, Heidelberg, Germany
3 Iganga General Hospital, Iganga, Uganda
4 Karolinska Institutet, Department of Public Health Sciences, Division of Global Health, Stockholm, Sweden
5 Makerere University, School of Public Health, Kampala, Uganda
6 Makerere University, Iganga/Mayuge Health and Demographic Surveillance System, Iganga, Uganda
§ Presenting author email: floscheibe@web.de
Electronic publication date: 2012 Oct 22
Publication date: 2012
Volume: 15
Issue: Suppl 3
Electronic Location ID: 18443-48
Copyright: © 2012 H.W.F. Neuhann et al.
Copyright year: 2012
License: This is an open-access article distributed under the terms of the Creative Commons Attribution License, which permits unrestricted use, distribution, and reproduction in any medium, provided the original work is properly cited.
License URL: http://creativecommons.org/licenses/by/2.0/

JOURNAL INFORMATION
==============================
NLM Title Abbreviation: J Int AIDS Soc
Journal ID: J Int AIDS Soc
NLM Journal ID: 101478566
Journal ID: JIAS

Journal of the International AIDS Society

EISSN: 1758-2652
Publisher: Wiley

ARTICLE INFORMATION
==============================
Article ID: 18443-49
Subjects: E53 - Decentralization of services

THAE0103: PMTCT decentralization does not assure optimal service delivery: revelations from successful individual-level tracking of HIV-positive mothers and their infants

Edmonds A. 1
Thompson D. 1
Okitolonda V. 2
Feinstein L. 1
Kawende B. 2
Behets F. 1 3
PTME Team §
1 The University of North Carolina at Chapel Hill, Epidemiology, Chapel Hill, United States
2 Kinshasa School of Public Health, Kinshasa, Democratic Republic of the Congo
3 The University of North Carolina at Chapel Hill, Medicine, Chapel Hill, United States
§ Presenting author email: aedmonds@email.unc.edu
Electronic publication date: 2012 Oct 22
Publication date: 2012
Volume: 15
Issue: Suppl 3
Electronic Location ID: 18443-49
Copyright: © 2012 PTME Team et al.
Copyright year: 2012
License: This is an open-access article distributed under the terms of the Creative Commons Attribution License, which permits unrestricted use, distribution, and reproduction in any medium, provided the original work is properly cited.
License URL: http://creativecommons.org/licenses/by/2.0/

JOURNAL INFORMATION
==============================
NLM Title Abbreviation: J Int AIDS Soc
Journal ID: J Int AIDS Soc
NLM Journal ID: 101478566
Journal ID: JIAS

Journal of the International AIDS Society

EISSN: 1758-2652
Publisher: Wiley

ARTICLE INFORMATION
==============================
Article ID: 18443-50
Subjects: E53 - Decentralization of services

THAE0104: Preventing mother-to-child HIV transmission through community-based approach in Nepal

Shafique N. 1 §
1 UNICEF, HIV and AIDS Section, Lalitpur, Nepal
§ Presenting author email: nbshafique@unicef.org
Electronic publication date: 2012 Oct 22
Publication date: 2012
Volume: 15
Issue: Suppl 3
Electronic Location ID: 18443-50
Copyright: © 2012 N. Shafique et al.
Copyright year: 2012
License: This is an open-access article distributed under the terms of the Creative Commons Attribution License, which permits unrestricted use, distribution, and reproduction in any medium, provided the original work is properly cited.
License URL: http://creativecommons.org/licenses/by/2.0/

JOURNAL INFORMATION
==============================
NLM Title Abbreviation: J Int AIDS Soc
Journal ID: J Int AIDS Soc
NLM Journal ID: 101478566
Journal ID: JIAS

Journal of the International AIDS Society

EISSN: 1758-2652
Publisher: Wiley

ARTICLE INFORMATION
==============================
Article ID: 18443-51
Subjects: E54 - Decentralization of services for ART, community care and continuity of care health services

WEPDE0106: Scaling-up access to antiretroviral treatment: a comparison of outcomes among HIV-positive children receiving treatment at mobile and hospital-based HIV clinics in rural Zambia

van Dijk J.H. 1 2 §
Moss W.J. 3
Sinywimaanzi P. 1
Hamangaba F. 1
Sutcliffe C.G. 3
1 Macha Research Trust, Clinical Research Department, Choma, Zambia
2 Erasmus University, Department of Immunology and Infectious Diseases, Rotterdam, Netherlands
3 Bloomberg School of Public Health, Johns Hopkins University, Department of Epidemiology, Baltimore, United States
§ Presenting author email: janneke.vandijk@miam.org.zm
Electronic publication date: 2012 Oct 22
Publication date: 2012
Volume: 15
Issue: Suppl 3
Electronic Location ID: 18443-51
Copyright: © 2012 J.H. van Dijk et al.
Copyright year: 2012
License: This is an open-access article distributed under the terms of the Creative Commons Attribution License, which permits unrestricted use, distribution, and reproduction in any medium, provided the original work is properly cited.
License URL: http://creativecommons.org/licenses/by/2.0/

JOURNAL INFORMATION
==============================
NLM Title Abbreviation: J Int AIDS Soc
Journal ID: J Int AIDS Soc
NLM Journal ID: 101478566
Journal ID: JIAS

Journal of the International AIDS Society

EISSN: 1758-2652
Publisher: Wiley

ARTICLE INFORMATION
==============================
Article ID: 18443-52
Subjects: E56 - Scaling-up methods and experiences for HIV programmes

WEAE0401: Sustaining quality while scaling-up adolescent ART: findings from Zimbabwe′s largest adolescent cohort

Shroufi A. 1 §
Dixon M. 2
Gunguwo H. 2
Nyathi M. 2
Ndlovu M. 2
Saint-Sauveur J.F. 1
Taziwa F. 1
Ndebele W. 2
Ferreyra C. 3
1 Medecins Sans Frontieres, Medical Department, Harare, Zimbabwe
2 Mpilo Central Hospital, Bulawayo, Zimbabwe
3 Medecins Sans Frontieres, Barcelona, Spain
§ Presenting author email: msfe-harare-epidem@barcelona.msf.org
Electronic publication date: 2012 Oct 22
Publication date: 2012
Volume: 15
Issue: Suppl 3
Electronic Location ID: 18443-52
Copyright: © 2012 A. Shroufi et al.
Copyright year: 2012
License: This is an open-access article distributed under the terms of the Creative Commons Attribution License, which permits unrestricted use, distribution, and reproduction in any medium, provided the original work is properly cited.
License URL: http://creativecommons.org/licenses/by/2.0/

JOURNAL INFORMATION
==============================
NLM Title Abbreviation: J Int AIDS Soc
Journal ID: J Int AIDS Soc
NLM Journal ID: 101478566
Journal ID: JIAS

Journal of the International AIDS Society

EISSN: 1758-2652
Publisher: Wiley

ARTICLE INFORMATION
==============================
Article ID: 18443-53
Subjects: E56 - Scaling-up methods and experiences for HIV programmes

THAE0105: Nothing for us without us: community led approaches towards successful implementation of HIV prevention programs: experiences from southern India

Anthony J. 1
Hugar V. 2
Francis Munjattu J. 1
Pal A. 1
Gyarappagari S. 1
Ghosh S.C. 3
Murugan S.K. 4
Fahim S.K. 5 §
1 India Health Action Trust, Technical Support Unit, Bangalore, India
2 Karnataka State AIDS Prevention Society, Targeted Interventions, Bangalore, India
3 National AIDS Control Organization, Targeted Intervention, New Delhi, India
4 University of Manitoba, Strategic Initiatives and Knowledge Translation, Bangalore, India
5 Karnataka State AIDS Prevention Society, Project Directorate, Bangalore, India
§ Presenting author email: john.anthony@ihat.in
Electronic publication date: 2012 Oct 22
Publication date: 2012
Volume: 15
Issue: Suppl 3
Electronic Location ID: 18443-53
Copyright: © 2012 J. Anthony et al.
Copyright year: 2012
License: This is an open-access article distributed under the terms of the Creative Commons Attribution License, which permits unrestricted use, distribution, and reproduction in any medium, provided the original work is properly cited.
License URL: http://creativecommons.org/licenses/by/2.0/

JOURNAL INFORMATION
==============================
NLM Title Abbreviation: J Int AIDS Soc
Journal ID: J Int AIDS Soc
NLM Journal ID: 101478566
Journal ID: JIAS

Journal of the International AIDS Society

EISSN: 1758-2652
Publisher: Wiley

ARTICLE INFORMATION
==============================
Article ID: 18443-54
Subjects: E57 - Integration of HIV services rather than stand-alone services, and services which bond the community care with the health facility services

WEPDE0206: HIV prevention through pharmacies network in Ukraine: improving access to services for injecting drug users and commercial sex workers

Naduta G. 1 §
1 ICF International HIV/AIDS Alliance in Ukraine, Policy and Partnership, Kyiv, Ukraine
§ Presenting author email: naduta@aidsalliance.org.ua
Electronic publication date: 2012 Oct 22
Publication date: 2012
Volume: 15
Issue: Suppl 3
Electronic Location ID: 18443-54
Copyright: © 2012 G. Naduta et al.
Copyright year: 2012
License: This is an open-access article distributed under the terms of the Creative Commons Attribution License, which permits unrestricted use, distribution, and reproduction in any medium, provided the original work is properly cited.
License URL: http://creativecommons.org/licenses/by/2.0/

JOURNAL INFORMATION
==============================
NLM Title Abbreviation: J Int AIDS Soc
Journal ID: J Int AIDS Soc
NLM Journal ID: 101478566
Journal ID: JIAS

Journal of the International AIDS Society

EISSN: 1758-2652
Publisher: Wiley

ARTICLE INFORMATION
==============================
Article ID: 18443-55
Subjects: E58 - Role of community organizations in linking people to HIV services and strengthening the health system

WEAE0204: Community-based adherence support associated with improved virological suppression in adults receiving antiretroviral treatment: five-year outcomes from a multicentre cohort study in South Africa

Fatti G. 1
Grimwood A. 1 §
Shea J. 2
1 Kheth'Impilo, Cape Town, South Africa
2 University of Cape Town, Child Health Unit, School of Child & Adolescent Health, Cape Town, South Africa
§ Presenting author email: ashraf.grimwood@khethimpilo.org
Electronic publication date: 2012 Oct 22
Publication date: 2012
Volume: 15
Issue: Suppl 3
Electronic Location ID: 18443-55
Copyright: © 2012 A. Grimwood et al.
Copyright year: 2012
License: This is an open-access article distributed under the terms of the Creative Commons Attribution License, which permits unrestricted use, distribution, and reproduction in any medium, provided the original work is properly cited.
License URL: http://creativecommons.org/licenses/by/2.0/

JOURNAL INFORMATION
==============================
NLM Title Abbreviation: J Int AIDS Soc
Journal ID: J Int AIDS Soc
NLM Journal ID: 101478566
Journal ID: JIAS

Journal of the International AIDS Society

EISSN: 1758-2652
Publisher: Wiley

ARTICLE INFORMATION
==============================
Article ID: 18443-56
Subjects: E58 - Role of community organizations in linking people to HIV services and strengthening the health system

THPDE0303: Effectiveness of rapid night-time HIV testing for men who have sex with men (MSM) in Kinshasa, Democratic Republic of Congo

Mbwolie Nsabala H. 1
Kapila G. 2
Makwelebi V. 2 §
Ndagano D. 3
1 Progrès Santé Sans Prix (PSSP), Kinshasa, Democratic Republic of the Congo
2 Chemonics International Inc., DRC Integrated HIV/AIDS Project (ProVIC), Kinshasa, Democratic Republic of the Congo
3 PATH, DRC Integrated HIV/AIDS Project (ProVIC), Kinshasa, Democratic Republic of the Congo
§ Presenting author email: vmindongo@provic.org
Electronic publication date: 2012 Oct 22
Publication date: 2012
Volume: 15
Issue: Suppl 3
Electronic Location ID: 18443-56
Copyright: © 2012 V. Makwelebi et al.
Copyright year: 2012
License: This is an open-access article distributed under the terms of the Creative Commons Attribution License, which permits unrestricted use, distribution, and reproduction in any medium, provided the original work is properly cited.
License URL: http://creativecommons.org/licenses/by/2.0/

JOURNAL INFORMATION
==============================
NLM Title Abbreviation: J Int AIDS Soc
Journal ID: J Int AIDS Soc
NLM Journal ID: 101478566
Journal ID: JIAS

Journal of the International AIDS Society

EISSN: 1758-2652
Publisher: Wiley

ARTICLE INFORMATION
==============================
Article ID: 18443-57
Subjects: E59 - Models for HIV service delivery in conflict and post-conflict settings

THPDE0202: Ensuring continuity of antiretroviral therapy among displaced populations during Ivorian post-election violence, 2011

Yiweza Tshipala D. 1 §
Cornier N. 2
Gounongbe M. 3
Petros G. 4
Assouan I. 3
Bilguissa D. 5
Spiegel P. 2
1 UNHCR, Public Health and HIV Section, Dakar, Senegal
2 UNHCR, Public Health and HIV Section, Geneva, Switzerland
3 UNHCR, Public Health and HIV Section, Abidjan, Cote D'Ivoire
4 UNHCR, Public Health and HIV Section, Monrovia, Liberia
5 UNHCR Consultant, Public Health and HIV Section, Paris, France
§ Presenting author email: yiweza@unhcr.org
Electronic publication date: 2012 Oct 22
Publication date: 2012
Volume: 15
Issue: Suppl 3
Electronic Location ID: 18443-57
Copyright: © 2012 D. Yiweza Tshipala et al.
Copyright year: 2012
License: This is an open-access article distributed under the terms of the Creative Commons Attribution License, which permits unrestricted use, distribution, and reproduction in any medium, provided the original work is properly cited.
License URL: http://creativecommons.org/licenses/by/2.0/

JOURNAL INFORMATION
==============================
NLM Title Abbreviation: J Int AIDS Soc
Journal ID: J Int AIDS Soc
NLM Journal ID: 101478566
Journal ID: JIAS

Journal of the International AIDS Society

EISSN: 1758-2652
Publisher: Wiley

ARTICLE INFORMATION
==============================
Article ID: 18443-58
Subjects: E59 - Models for HIV service delivery in conflict and post-conflict settings

THPDE0203: The challenge of maintaining continuum of care and support to PLHIV in health facilities located in military conflict zones in Ivory Coast

Doumbouya B. 1
Zaho M. 2
Adouko S. 1
Komena E. 3
Mensah-Maïka B. 4
Touré S. 5
1 ACONDA VS CI, Public Health, Abidjan, Cote D'Ivoire
2 ACONDA VS CI, PWP, Abidjan, Cote D'Ivoire
3 ACONDA VS CI, Care and Support, Abidjan, Cote D'Ivoire
4 ACONDA VS CI, Prevention, Abidjan, Cote D'Ivoire
5 ACONDA VS CI, Poject Executive Direction, Abidjan, Cote D'Ivoire
Electronic publication date: 2012 Oct 22
Publication date: 2012
Volume: 15
Issue: Suppl 3
Electronic Location ID: 18443-58
Copyright: © 2012 B. Doumbouya et al.
Copyright year: 2012
License: This is an open-access article distributed under the terms of the Creative Commons Attribution License, which permits unrestricted use, distribution, and reproduction in any medium, provided the original work is properly cited.
License URL: http://creativecommons.org/licenses/by/2.0/

JOURNAL INFORMATION
==============================
NLM Title Abbreviation: J Int AIDS Soc
Journal ID: J Int AIDS Soc
NLM Journal ID: 101478566
Journal ID: JIAS

Journal of the International AIDS Society

EISSN: 1758-2652
Publisher: Wiley

ARTICLE INFORMATION
==============================
Article ID: 18443-59
Subjects: E60 - Micro-finance programmes

FRAE0105: Small scale income generating initiatives mitigate the socio-economic vulnerability of HIV households in Bangladesh

Purification N. 1 §
1 Notun Jibon Shomaj Kollan Shongstha (NJSKS), Dhaka, Bangladesh
§ Presenting author email: npurification@gmail.com
Electronic publication date: 2012 Oct 22
Publication date: 2012
Volume: 15
Issue: Suppl 3
Electronic Location ID: 18443-59
Copyright: © 2012 N. Purification et al.
Copyright year: 2012
License: This is an open-access article distributed under the terms of the Creative Commons Attribution License, which permits unrestricted use, distribution, and reproduction in any medium, provided the original work is properly cited.
License URL: http://creativecommons.org/licenses/by/2.0/

JOURNAL INFORMATION
==============================
NLM Title Abbreviation: J Int AIDS Soc
Journal ID: J Int AIDS Soc
NLM Journal ID: 101478566
Journal ID: JIAS

Journal of the International AIDS Society

EISSN: 1758-2652
Publisher: Wiley

ARTICLE INFORMATION
==============================
Article ID: 18443-60
Subjects: E63 - Economic evaluation of prevention, treatment, care and mitigation programmes, adherence schemes, sustainability of programmes and financial strengthening initiatives

MOAE0201: Cost and efficiency analysis of the Avahan HIV prevention programme for high risk groups in India

Chandrashekar S. 1 §
Vassal A. 2
Shetty G. 3
Alary M. 4
Vickerman P. 2
1 Dept of Epidemiology, St. John's Research Institute, Bangalore, India
2 London School of Hygiene and Tropical MedicineGlobal Health and Development, London, United Kingdom
3 Karnataka Helath Promotion Trust, Bangalore, India
4 Centre Hospitalier affilie Universitaire de QuebecPopulation Health Research, Quebec, Canada
§ Presenting author email: sudhashreec@yahoo.co.in
Electronic publication date: 2012 Oct 22
Publication date: 2012
Volume: 15
Issue: Suppl 3
Electronic Location ID: 18443-60
Copyright: © 2012 S. Chandrashekar et al.
Copyright year: 2012
License: This is an open-access article distributed under the terms of the Creative Commons Attribution License, which permits unrestricted use, distribution, and reproduction in any medium, provided the original work is properly cited.
License URL: http://creativecommons.org/licenses/by/2.0/

JOURNAL INFORMATION
==============================
NLM Title Abbreviation: J Int AIDS Soc
Journal ID: J Int AIDS Soc
NLM Journal ID: 101478566
Journal ID: JIAS

Journal of the International AIDS Society

EISSN: 1758-2652
Publisher: Wiley

ARTICLE INFORMATION
==============================
Article ID: 18443-61
Subjects: E63 - Economic evaluation of prevention, treatment, care and mitigation programmes, adherence schemes, sustainability of programmes and financial strengthening initiatives

MOAE0204: Increasing investment in syringe exchange is cost-saving HIV prevention: modeling hypothetical syringe coverage levels in the United States

Nguyen T.Q. 1 §
Weir B.W. 1
Pinkerton S.D. 2
Des Jarlais D.C. 3
Holtgrave D. 1
1 Department of Health, Behavior and Society, Johns Hopkins Bloomberg School of Public Health, Baltimore, United States
2 Department of Psychiatry and Behavioral Medicine, Medical College of Wisconsin, Milwaukee, United States
3 Beth Israel Medical Center and North American Syringe Exchange Network, New York, United States
§ Presenting author email: tqnguyen@jhsph.edu
Electronic publication date: 2012 Oct 22
Publication date: 2012
Volume: 15
Issue: Suppl 3
Electronic Location ID: 18443-61
Copyright: © 2012 T.Q. Nguyen et al.
Copyright year: 2012
License: This is an open-access article distributed under the terms of the Creative Commons Attribution License, which permits unrestricted use, distribution, and reproduction in any medium, provided the original work is properly cited.
License URL: http://creativecommons.org/licenses/by/2.0/

JOURNAL INFORMATION
==============================
NLM Title Abbreviation: J Int AIDS Soc
Journal ID: J Int AIDS Soc
NLM Journal ID: 101478566
Journal ID: JIAS

Journal of the International AIDS Society

EISSN: 1758-2652
Publisher: Wiley

ARTICLE INFORMATION
==============================
Article ID: 18443-62
Subjects: E63 - Economic evaluation of prevention, treatment, care and mitigation programmes, adherence schemes, sustainability of programmes and financial strengthening initiatives

FRAE0101: What does it cost to raise an orphan? A comparison of OVC costs in Ethiopia and Botswana

Telake D. 1
Formson C. 2
Forsythe S. 3 §
1 Independent Consultant, London, Canada
2 Independent Consultant, Gaborone, Botswana
3 Futures Institute, Fountain Hills, United States
§ Presenting author email: sforsythe@futuresinstitute.org
Electronic publication date: 2012 Oct 22
Publication date: 2012
Volume: 15
Issue: Suppl 3
Electronic Location ID: 18443-62
Copyright: © 2012 S. Forsythe et al.
Copyright year: 2012
License: This is an open-access article distributed under the terms of the Creative Commons Attribution License, which permits unrestricted use, distribution, and reproduction in any medium, provided the original work is properly cited.
License URL: http://creativecommons.org/licenses/by/2.0/

JOURNAL INFORMATION
==============================
NLM Title Abbreviation: J Int AIDS Soc
Journal ID: J Int AIDS Soc
NLM Journal ID: 101478566
Journal ID: JIAS

Journal of the International AIDS Society

EISSN: 1758-2652
Publisher: Wiley

ARTICLE INFORMATION
==============================
Article ID: 18443-63
Subjects: E63 - Economic evaluation of prevention, treatment, care and mitigation programmes, adherence schemes, sustainability of programmes and financial strengthening initiatives

FRAE0103: Economic evaluation of the national program to prevent mother-to-child transmission of HIV in Ghana

El-Adas A. 1 §
Amenyah R. 2
Koleros A. 3
Akwei Addo N. 4
Asante F. 5
Wondergem P. 6
Atuahene K. 7
Asante K. 4
1 Ghana AIDS Commission, Accra, Ghana
2 Ghana AIDS Commission, Technical Services Directorate, Accra, Ghana
3 Futures Group/Health Policy Project, London, United Kingdom
4 National AIDS and STD Control Program, Accra, Ghana
5 University of Ghana, Institute of Statistical, Social, and Economic Research, Accra, Ghana
6 USAID Ghana, Accra, Ghana
7 Ghana AIDS Commission, Research, Monitoring and Evaluation Division, Accra, Ghana
§ Presenting author email: aeladas@ghanaids.gov.gh
Electronic publication date: 2012 Oct 22
Publication date: 2012
Volume: 15
Issue: Suppl 3
Electronic Location ID: 18443-63
Copyright: © 2012 A. El-Adas et al.
Copyright year: 2012
License: This is an open-access article distributed under the terms of the Creative Commons Attribution License, which permits unrestricted use, distribution, and reproduction in any medium, provided the original work is properly cited.
License URL: http://creativecommons.org/licenses/by/2.0/

JOURNAL INFORMATION
==============================
NLM Title Abbreviation: J Int AIDS Soc
Journal ID: J Int AIDS Soc
NLM Journal ID: 101478566
Journal ID: JIAS

Journal of the International AIDS Society

EISSN: 1758-2652
Publisher: Wiley

ARTICLE INFORMATION
==============================
Article ID: 18443-64
Subjects: E64 - Sustainability of ART programmes and adherence in developing countries

MOAE0205: Transition from stavudine to tenofovir and zidovudine for first-line treatment of HIV/AIDS in low- and middle-income countries

Dancel E. 1
Ashigbie P. 1 §
1 Department of Family Medicine, Boston University School of Medicine, Boston, United States
§ Presenting author email: ellendancel@gmail.com
Electronic publication date: 2012 Oct 22
Publication date: 2012
Volume: 15
Issue: Suppl 3
Electronic Location ID: 18443-64
Copyright: © 2012 P. Ashigbie et al.
Copyright year: 2012
License: This is an open-access article distributed under the terms of the Creative Commons Attribution License, which permits unrestricted use, distribution, and reproduction in any medium, provided the original work is properly cited.
License URL: http://creativecommons.org/licenses/by/2.0/

JOURNAL INFORMATION
==============================
NLM Title Abbreviation: J Int AIDS Soc
Journal ID: J Int AIDS Soc
NLM Journal ID: 101478566
Journal ID: JIAS

Journal of the International AIDS Society

EISSN: 1758-2652
Publisher: Wiley

ARTICLE INFORMATION
==============================
Article ID: 18443-65
Subjects: E65 - Economic strengthening initiatives (including micro-finance, health insurance, etc.)

WEAE0402: Self-esteem, self-efficacy and hope among vulnerable adolescents affected by HIV participating in community-based savings and lending groups in rural Nyanga district, Zimbabwe

Senefeld S. 1
Miller C. 1 §
Mgugu D. 2
Mavise G. 2
Rowe W.-A. 1
1 Catholic Relief Services (CRS), Baltimore, United States
2 Catholic Relief Services Zimbabwe, Harare, Zimbabwe
§ Presenting author email: carrie.miller@crs.org
Electronic publication date: 2012 Oct 22
Publication date: 2012
Volume: 15
Issue: Suppl 3
Electronic Location ID: 18443-65
Copyright: © 2012 C. Miller et al.
Copyright year: 2012
License: This is an open-access article distributed under the terms of the Creative Commons Attribution License, which permits unrestricted use, distribution, and reproduction in any medium, provided the original work is properly cited.
License URL: http://creativecommons.org/licenses/by/2.0/

JOURNAL INFORMATION
==============================
NLM Title Abbreviation: J Int AIDS Soc
Journal ID: J Int AIDS Soc
NLM Journal ID: 101478566
Journal ID: JIAS

Journal of the International AIDS Society

EISSN: 1758-2652
Publisher: Wiley

ARTICLE INFORMATION
==============================
Article ID: 18443-66
Subjects: E66 - Consequences of test-and-treat programmes on investments in human capital and on household finances/wealth

THAE0102: Improved employment and children's education outcomes in households of HIV-positive adults with high CD4 counts: evidence from a community-wide health campaign in Uganda

Thirumurthy H. 1 §
Chamie G. 2 3
Kabami J. 3
Jain V. 2 3
Clark T. 2 3
Kwarisiima D. 4
Geng E. 2 3
Petersen M.L. 5
Kamya M.R. 3 6
Charlebois E.D. 2 7
Havlir D.V. 2 3
the SEARCH Collaboration
1 Department of Health Policy and Management, University of North Carolina at Chapel Hill, Chapel Hill, United States
2 Department of Medicine, University of California, San Francisco, HIV/AIDS Division, San Francisco, United States
3 Makerere University-University of California, San Francisco (MU-UCSF) Research Collaboration, Kampala, Uganda
4 Mulago-Mbarara Joint AIDS Program, Mbarara, Uganda
5 University of California at Berkeley, School of Public Health, Berkeley, United States
6 Makerere University Medical School, Kampala, Uganda
7 Center for AIDS Prevention Studies, University of California, San Francisco, San Francisco, United States
§ Presenting author email: harsha@unc.edu
Electronic publication date: 2012 Oct 22
Publication date: 2012
Volume: 15
Issue: Suppl 3
Electronic Location ID: 18443-66
Copyright: © 2012 H. Thirumurthy et al.
Copyright year: 2012
License: This is an open-access article distributed under the terms of the Creative Commons Attribution License, which permits unrestricted use, distribution, and reproduction in any medium, provided the original work is properly cited.
License URL: http://creativecommons.org/licenses/by/2.0/

JOURNAL INFORMATION
==============================
NLM Title Abbreviation: J Int AIDS Soc
Journal ID: J Int AIDS Soc
NLM Journal ID: 101478566
Journal ID: JIAS

Journal of the International AIDS Society

EISSN: 1758-2652
Publisher: Wiley

ARTICLE INFORMATION
==============================
Article ID: 18443-67
Subjects: E66 - Consequences of test-and-treat programmes on investments in human capital and on household finances/wealth

TULBE05: Dramatic increases in population life expectancy and the economic value of ART in rural South Africa

Bor J. 1 2 §
Herbst A.J. 2
Newell M.-L. 2 3
Bärnighausen T. 1 2
1 Harvard School of Public Health, Global Health and Population, Boston, United States
2 Africa Centre for Health and Population Studies, University of KwaZulu-Natal, Mtubatuba, South Africa
3 Centre for Paediatric Epidemiology and Biostatistics, University College of London Institute of Child Health, London, United Kingdom
§ Presenting author email: jbor@hsph.harvard.edu
Electronic publication date: 2012 Oct 22
Publication date: 2012
Volume: 15
Issue: Suppl 3
Electronic Location ID: 18443-67
Copyright: © 2012 J. Bor et al.
Copyright year: 2012
License: This is an open-access article distributed under the terms of the Creative Commons Attribution License, which permits unrestricted use, distribution, and reproduction in any medium, provided the original work is properly cited.
License URL: http://creativecommons.org/licenses/by/2.0/

JOURNAL INFORMATION
==============================
NLM Title Abbreviation: J Int AIDS Soc
Journal ID: J Int AIDS Soc
NLM Journal ID: 101478566
Journal ID: JIAS

Journal of the International AIDS Society

EISSN: 1758-2652
Publisher: Wiley

ARTICLE INFORMATION
==============================
Article ID: 18443-68
Subjects: E66 - Consequences of test-and-treat programmes on investments in human capital and on household finances/wealth

MOPDE0206: The impact of antiretroviral therapy on the social, economic and working conditions of patients with HIV in Malawi

Orlando S. 1 §
Alumando E.S. 2
Marazzi M.C. 3
Germano P. 4
Guidotti G. 5
Gennaro E. 6
Ciccacci F. 7
Palombi L. 8
1 Community of Sant'Egidio, DREAM Program, Rome, Italy
2 University of Rome ‘Tor Vergata’, Hygiene, Blantyre, Malawi
3 Education Sciences, L.U.M.S.A. University, Rome, Italy
4 INMI ‘Lazzaro Spallanzani’ I.R.C.C.S., Rome, Italy
5 University of Pisa, Pisa, Italy
6 Università Gabriele D'Annunzio Chieti Pescara, Chieti, Italy
7 University of Rome ‘La Sapienza’, Rome, Italy
8 University of Rome ‘Tor Vergata’, Hygiene, Rome, Italy
§ Presenting author email: stefano.orlando@dreameurope.org
Electronic publication date: 2012 Oct 22
Publication date: 2012
Volume: 15
Issue: Suppl 3
Electronic Location ID: 18443-68
Copyright: © 2012 S. Orlando et al.
Copyright year: 2012
License: This is an open-access article distributed under the terms of the Creative Commons Attribution License, which permits unrestricted use, distribution, and reproduction in any medium, provided the original work is properly cited.
License URL: http://creativecommons.org/licenses/by/2.0/

JOURNAL INFORMATION
==============================
NLM Title Abbreviation: J Int AIDS Soc
Journal ID: J Int AIDS Soc
NLM Journal ID: 101478566
Journal ID: JIAS

Journal of the International AIDS Society

EISSN: 1758-2652
Publisher: Wiley

ARTICLE INFORMATION
==============================
Article ID: 18443-69
Subjects: E66 - Consequences of test-and-treat programmes on investments in human capital and on household finances/wealth

WEPDE0201: Does condom use affect the earnings of commercial sex workers? New evidence from a survey of female sex workers in India

Mondal S. 1 §
Gupta I. 1
1 Institute of Economic Growth, Delhi, India
§ Presenting author email: kumar.swadhin@gmail.com
Electronic publication date: 2012 Oct 22
Publication date: 2012
Volume: 15
Issue: Suppl 3
Electronic Location ID: 18443-69
Copyright: © 2012 S. Mondal et al.
Copyright year: 2012
License: This is an open-access article distributed under the terms of the Creative Commons Attribution License, which permits unrestricted use, distribution, and reproduction in any medium, provided the original work is properly cited.
License URL: http://creativecommons.org/licenses/by/2.0/

JOURNAL INFORMATION
==============================
NLM Title Abbreviation: J Int AIDS Soc
Journal ID: J Int AIDS Soc
NLM Journal ID: 101478566
Journal ID: JIAS

Journal of the International AIDS Society

EISSN: 1758-2652
Publisher: Wiley

ARTICLE INFORMATION
==============================
Article ID: 18443-70
Subjects: E68 - Investment frameworks and models in a period and in settings of decreasing financial resources.

MOAE0203: Company-level ART provision to employees is cost saving: a modelled cost-benefit analysis of the impact of HIV and antiretroviral treatment in a mining workforce in South Africa

Meyer-Rath G. 1 2 3
Pienaar J. 4
Brink B. 5
van Zyl A. 6
Muirhead D. 6
Grant A. 7
Churchyard G. 6
Watts C. 2
Vickerman P. 8 §
1 Boston University Center for Global Health and Development, Boston, United States
2 London School of Hygiene and Tropical Medicine, Department of Health Services Research and Policy, London, United Kingdom
3 University of the Witwatersrand, Health Economics and Epidemiology Research Office (HE2RO), Johannesburg, South Africa
4 Highveld Hospital, Thermal Coal, Emhalahleni, South Africa
5 Anglo American, Johannesburg, South Africa
6 The Aurum Institute, Johannesburg, South Africa
7 Department of Clinical Research, London School of Hygiene and Tropical Medicine, London, United Kingdom
8 London School of Hygiene and Tropical Medicine, Department of Global Health and Development, London, United Kingdom
§ Presenting author email: peter.vickerman@lshtm.ac.uk
Electronic publication date: 2012 Oct 22
Publication date: 2012
Volume: 15
Issue: Suppl 3
Electronic Location ID: 18443-70
Copyright: © 2012 P. Vickerman et al.
Copyright year: 2012
License: This is an open-access article distributed under the terms of the Creative Commons Attribution License, which permits unrestricted use, distribution, and reproduction in any medium, provided the original work is properly cited.
License URL: http://creativecommons.org/licenses/by/2.0/

JOURNAL INFORMATION
==============================
NLM Title Abbreviation: J Int AIDS Soc
Journal ID: J Int AIDS Soc
NLM Journal ID: 101478566
Journal ID: JIAS

Journal of the International AIDS Society

EISSN: 1758-2652
Publisher: Wiley

ARTICLE INFORMATION
==============================
Article ID: 18443-71
Subjects: E70 - Cross-national comparisons

WEAE0403: The state of the national response to prevent HIV among young people: a review of national reporting in 20 high-prevalence countries

Birdthistle I. 1
Dringus S. 1
Knight L. 2
Yankah E. 2
Idele P. 3
Suzuki C. 3
Nguyen L. 3
Kasedde S. 4 §
1 London School of Hygiene & Tropical Medicine, Epidemiology & Population Health, London, United Kingdom
2 Consultant, London, United Kingdom
3 UNICEF, New York, United States
4 UNICEF, New York, United Kingdom
§ Presenting author email: isolde.birdthistle@lshtm.ac.uk
Electronic publication date: 2012 Oct 22
Publication date: 2012
Volume: 15
Issue: Suppl 3
Electronic Location ID: 18443-71
Copyright: © 2012 S. Kasedde et al.
Copyright year: 2012
License: This is an open-access article distributed under the terms of the Creative Commons Attribution License, which permits unrestricted use, distribution, and reproduction in any medium, provided the original work is properly cited.
License URL: http://creativecommons.org/licenses/by/2.0/

JOURNAL INFORMATION
==============================
NLM Title Abbreviation: J Int AIDS Soc
Journal ID: J Int AIDS Soc
NLM Journal ID: 101478566
Journal ID: JIAS

Journal of the International AIDS Society

EISSN: 1758-2652
Publisher: Wiley

ARTICLE INFORMATION
==============================
Article ID: 18443-72
Subjects: E72 - Sustainability of financing and programmes

MOAE0302: Sustainability of HIV programs: lessons learned from sustainability analyses in four countries

Katz I. 1 §
Wong W. 1
Glandon D. 1
Kargbo B. 2
Ombam R. 3
Singh S. 4
Osika J. 1
1 Abt Associates, Health Systems 20/20, Bethesda, United States
2 National AIDS Secretariat, Freetown, Sierra Leone
3 National AIDS Control Council, Nairobi, Kenya
4 National AIDS Programme Secretariat, Georgetown, Guyana
§ Presenting author email: katz@cantab.net
Electronic publication date: 2012 Oct 22
Publication date: 2012
Volume: 15
Issue: Suppl 3
Electronic Location ID: 18443-72
Copyright: © 2012 I. Katz et al.
Copyright year: 2012
License: This is an open-access article distributed under the terms of the Creative Commons Attribution License, which permits unrestricted use, distribution, and reproduction in any medium, provided the original work is properly cited.
License URL: http://creativecommons.org/licenses/by/2.0/

JOURNAL INFORMATION
==============================
NLM Title Abbreviation: J Int AIDS Soc
Journal ID: J Int AIDS Soc
NLM Journal ID: 101478566
Journal ID: JIAS

Journal of the International AIDS Society

EISSN: 1758-2652
Publisher: Wiley

ARTICLE INFORMATION
==============================
Article ID: 18443-73
Subjects: E72 - Sustainability of financing and programmes

WEAE0105: Affordability of HIV/AIDS treatment in developing countries: an analysis of ARV drug price determinants

Sagaon Teyssier L. 1 2 3
Arrighi Y. 1 3 4
Dongmo Nguimfack B. 5
Moatti J.-P. 1 2 3 §
1 INSERM, U912 (SESSTIM), Marseille, France
2 Aix Marseille Univ, IRD, UMR-S912, Marseille, France
3 ORS PACA, Observatoire Régional de la Santé Provence Alpes Côte d'Azur, Marseille, France
4 GREQAM, Groupement de Recherche en Economie Quantitative d'Aix-Marseille, Marseille, France
5 WHO, World Health OrganizationGeneva, Switzerland
§ Presenting author email: jean-paul.moatti@inserm.fr
Electronic publication date: 2012 Oct 22
Publication date: 2012
Volume: 15
Issue: Suppl 3
Electronic Location ID: 18443-73
Copyright: © 2012 J.-P. Moatti et al.
Copyright year: 2012
License: This is an open-access article distributed under the terms of the Creative Commons Attribution License, which permits unrestricted use, distribution, and reproduction in any medium, provided the original work is properly cited.
License URL: http://creativecommons.org/licenses/by/2.0/

JOURNAL INFORMATION
==============================
NLM Title Abbreviation: J Int AIDS Soc
Journal ID: J Int AIDS Soc
NLM Journal ID: 101478566
Journal ID: JIAS

Journal of the International AIDS Society

EISSN: 1758-2652
Publisher: Wiley

ARTICLE INFORMATION
==============================
Article ID: 18443-74
Subjects: E72 - Sustainability of financing and programmes

TULBE06: Scenario-based cost projections for PEPFAR resource requirements for the ART program in Ethiopia from FY2011-FY2015

Ali S.A. 1 §
Menzies N. 2
Kenyon T. 1
Melaku Z. 3
Gadisa T. 3
Graham B. 4
Feleke G. 4
Zewdu S. 5
Makonnen M. 6
Mattanovich D. 7
Mamo Y. 7
1 United States Centers for Disease Control & Prevention (CDC)-Ethiopia, Addis Ababa, Ethiopia
2 ICF Macro, Atlanta, United States
3 Columbia University International Center for AIDS Care and Treatment Program (CU ICAP)-Ethiopia, Addis Ababa, Ethiopia
4 University of Washington International Training and Education Center for Health (I TECH)-Ethiopia, Addis Ababa, Ethiopia
5 Johns Hopkins University Technical Support for The Ethiopian HIV/AIDS ART Initiative (JHU TSEHAI), Addis Ababa, Ethiopia
6 Catholic Relief Services/AIDSRelief Ethiopia, Addis Ababa, Ethiopia
7 University of California-San Diego (UCSD)-Ethiopia, Addis Ababa, Ethiopia
§ Presenting author email: ahmeds@et.cdc.gov
Electronic publication date: 2012 Oct 22
Publication date: 2012
Volume: 15
Issue: Suppl 3
Electronic Location ID: 18443-74
Copyright: © 2012 S.A. Ali et al.
Copyright year: 2012
License: This is an open-access article distributed under the terms of the Creative Commons Attribution License, which permits unrestricted use, distribution, and reproduction in any medium, provided the original work is properly cited.
License URL: http://creativecommons.org/licenses/by/2.0/

JOURNAL INFORMATION
==============================
NLM Title Abbreviation: J Int AIDS Soc
Journal ID: J Int AIDS Soc
NLM Journal ID: 101478566
Journal ID: JIAS

Journal of the International AIDS Society

EISSN: 1758-2652
Publisher: Wiley

ARTICLE INFORMATION
==============================
Article ID: 18443-75
Subjects: E73 - Measuring efficiency and effectiveness of national AIDS programmes

MOAE0303: Spending on ART by provinces in South Africa: trends, cost drivers, (in)efficiencies and sustainability

Simelela N. 1
Senabe S. 2
Sozi C. 3
Damisoni H. 3
Guthrie T. 4
1 President's Office, South Africa, Pretoria, South Africa
2 Department of Public Service and Administration, Pretoria, South Africa
3 UNAIDS, Pretoria, South Africa
4 Centre for Economic Governance and AIDS in Africa (CEGAA), Cape Town, South Africa
Electronic publication date: 2012 Oct 22
Publication date: 2012
Volume: 15
Issue: Suppl 3
Electronic Location ID: 18443-75
Copyright: © 2012 N. Simelela et al.
Copyright year: 2012
License: This is an open-access article distributed under the terms of the Creative Commons Attribution License, which permits unrestricted use, distribution, and reproduction in any medium, provided the original work is properly cited.
License URL: http://creativecommons.org/licenses/by/2.0/

JOURNAL INFORMATION
==============================
NLM Title Abbreviation: J Int AIDS Soc
Journal ID: J Int AIDS Soc
NLM Journal ID: 101478566
Journal ID: JIAS

Journal of the International AIDS Society

EISSN: 1758-2652
Publisher: Wiley

ARTICLE INFORMATION
==============================
Article ID: 18443-76
Subjects: E74 - Ethics of operations research, HIV programme implementation and economic evaluation

WEAE0303: Mobile phone adherence support for antiretroviral therapy: what would it cost the National AIDS Control Program in India?

Rodrigues R. 1 2
Shet A. 2 3
Swaroop N. 4
Shastri S. 5
Bogg L. 6
De Costa A. 2
1 St John's National Academy of Health Sciences, Community Health, Bangalore, India
2 Karolinska Institutet, Global Health, Stockholm, Sweden
3 St. John's National Academy of Health Sciences, Pediatrics, Bangalore, India
4 St John's National Academy of Health SciencesHIVIND Project, Bangalore, India
5 Karnataka State AIDS Prevention Society, Bangalore, India
6 School of Sustainable Development of Society and Technology, Stockholm, Sweden
Electronic publication date: 2012 Oct 22
Publication date: 2012
Volume: 15
Issue: Suppl 3
Electronic Location ID: 18443-76
Copyright: © 2012 R. Rodrigues et al.
Copyright year: 2012
License: This is an open-access article distributed under the terms of the Creative Commons Attribution License, which permits unrestricted use, distribution, and reproduction in any medium, provided the original work is properly cited.
License URL: http://creativecommons.org/licenses/by/2.0/

JOURNAL INFORMATION
==============================
NLM Title Abbreviation: J Int AIDS Soc
Journal ID: J Int AIDS Soc
NLM Journal ID: 101478566
Journal ID: JIAS

Journal of the International AIDS Society

EISSN: 1758-2652
Publisher: Wiley

ARTICLE INFORMATION
==============================
Article ID: 18443-77
Subjects: E75 - Use of operations research to support the response to HIV prevention and treatment

MOPDE0202: Factors associated with non-utilisation of antenatal care services by women delivering with an unknown HIV status at four poly-clinics in Chitungwiza, Zimbabwe

Makani V. 1 §
Rusakaniko S. 1
Angela A. 2
Makunike -Chikwinya B. 3
Shetty A. 4
Chandisarewa W. 1
ZAPP-UZ-TEAM Study Group
1 University of Zimbabwe College of Health Sciences, Community Medicine, Harare, Zimbabwe
2 Ministry of Health and Child Welfare, AIDS and TB Unit, Harare, Zimbabwe
3 Elizabeth Glaser Pediatric AIDS Foundation, Zimbabwe Country Office, Harare, Zimbabwe
4 Wake Forrest University Health Sciences, Pediatrics HIV Programme, Winston-Salem, United States
§ Presenting author email: vimbai@zappuz.co.zw
Electronic publication date: 2012 Oct 22
Publication date: 2012
Volume: 15
Issue: Suppl 3
Electronic Location ID: 18443-77
Copyright: © 2012 V. Makani et al.
Copyright year: 2012
License: This is an open-access article distributed under the terms of the Creative Commons Attribution License, which permits unrestricted use, distribution, and reproduction in any medium, provided the original work is properly cited.
License URL: http://creativecommons.org/licenses/by/2.0/

JOURNAL INFORMATION
==============================
NLM Title Abbreviation: J Int AIDS Soc
Journal ID: J Int AIDS Soc
NLM Journal ID: 101478566
Journal ID: JIAS

Journal of the International AIDS Society

EISSN: 1758-2652
Publisher: Wiley

ARTICLE INFORMATION
==============================
Article ID: 18443-78
Subjects: E77 - Operations research making a difference in programme performance

WEAE0203: Risk factors and true outcomes of children lost to follow-up from antiretroviral therapy in Lilongwe, Malawi

Ardura Garcia C. 1
Tweya H. 2
Feldacker C. 2
Phiri S. 2
Weigel R. 3 4
1 Liverpool School of Topical Medicine, Liverpool, United Kingdom
2 Lighthouse Trust Clinic, Kamuzu Central Hospital, Lilongwe, Malawi
3 Liverpool School of Topical Medicine, Disease Control Strategy Group, Liverpool, United Kingdom
4 Lighthouse Trust, Kamuzu Central Hospital, Lilongwe, Malawi
Electronic publication date: 2012 Oct 22
Publication date: 2012
Volume: 15
Issue: Suppl 3
Electronic Location ID: 18443-78
Copyright: © 2012 H. Tweya et al.
Copyright year: 2012
License: This is an open-access article distributed under the terms of the Creative Commons Attribution License, which permits unrestricted use, distribution, and reproduction in any medium, provided the original work is properly cited.
License URL: http://creativecommons.org/licenses/by/2.0/

JOURNAL INFORMATION
==============================
NLM Title Abbreviation: J Int AIDS Soc
Journal ID: J Int AIDS Soc
NLM Journal ID: 101478566
Journal ID: JIAS

Journal of the International AIDS Society

EISSN: 1758-2652
Publisher: Wiley

ARTICLE INFORMATION
==============================
Article ID: 18443-79
Subjects: E80 - Strategies for phasing-in viral load in resource-limited settings

TUPDE0206: Use of viral load testing in resource-limited settings to confirm treatment failure in children: experience from Nyanza Province, western Kenya

Muttai H. 1 §
Wanjiku L. 2
Gichangi A. 2
Miruka F. 1
Ayuaya T. 3
Soti D. 4
Zeh C. 1
Ackers M. 5
1 Centres for Disease Control and Prevention (CDC), Division of Global HIV/AIDS, Kisumu, Kenya
2 Centres for Disease Control and Prevention (CDC), Division of Global HIV/AIDS, Nairobi, Kenya
3 KEMRI/CDC Research and Public Health Collaboration, HISS, Kisumu, Kenya
4 Ministry of Health, Kisumu, Kenya
5 Centres for Disease Control and Prevention (CDC), Atlanta, United States
§ Presenting author email: hmuttai@ke.cdc.gov
Electronic publication date: 2012 Oct 22
Publication date: 2012
Volume: 15
Issue: Suppl 3
Electronic Location ID: 18443-79
Copyright: © 2012 H. Muttai et al.
Copyright year: 2012
License: This is an open-access article distributed under the terms of the Creative Commons Attribution License, which permits unrestricted use, distribution, and reproduction in any medium, provided the original work is properly cited.
License URL: http://creativecommons.org/licenses/by/2.0/

JOURNAL INFORMATION
==============================
NLM Title Abbreviation: J Int AIDS Soc
Journal ID: J Int AIDS Soc
NLM Journal ID: 101478566
Journal ID: JIAS

Journal of the International AIDS Society

EISSN: 1758-2652
Publisher: Wiley

ARTICLE INFORMATION
==============================
Article ID: 18443-80
Subjects: E81 - Use of viral load in prevention of mother-to-child transmission (PMTCT) programmes

TUPDE0205: Routine viral load testing among pregnant HIV-positive women on antiretroviral therapy: implications for prevention, Nyanza province, Kenya, 2011

Nganga L. 1 §
Muttai H. 1
Anthony G. 1
Miruka F. 1
Soti D. 2
Okonji J. 3
Ackers M. 4
1 Centers for Disease Control and Prevention, Nairobi, Kenya
2 Kenya Ministry of Public Health and Sanitation, Nairobi, Kenya
3 Kenya Medical Research Institute, Nairobi, Kenya
4 Centers for Disease Control and Prevention, Atlanta, United States
§ Presenting author email: lnganga@ke.cdc.gov
Electronic publication date: 2012 Oct 22
Publication date: 2012
Volume: 15
Issue: Suppl 3
Electronic Location ID: 18443-80
Copyright: © 2012 L. Nganga et al.
Copyright year: 2012
License: This is an open-access article distributed under the terms of the Creative Commons Attribution License, which permits unrestricted use, distribution, and reproduction in any medium, provided the original work is properly cited.
License URL: http://creativecommons.org/licenses/by/2.0/

JOURNAL INFORMATION
==============================
NLM Title Abbreviation: J Int AIDS Soc
Journal ID: J Int AIDS Soc
NLM Journal ID: 101478566
Journal ID: JIAS

Journal of the International AIDS Society

EISSN: 1758-2652
Publisher: Wiley

ARTICLE INFORMATION
==============================
Article ID: 18443-81
Subjects: E82 - Operations research in the use of laboratory monitoring, other than viral load

TUPDE0202: Assessment of laboratory test utilization for HIV/AIDS care in ART clinics of Malawi

Palchaudhuri S. 1 §
Tweya H. 2
Hosseinipour M. 1
1 UNC Project-Malawi, Lilongwe, Malawi
2 Lighthouse Trust Clinic, Lilongwe, Malawi
§ Presenting author email: sonalipc@umich.edu
Electronic publication date: 2012 Oct 22
Publication date: 2012
Volume: 15
Issue: Suppl 3
Electronic Location ID: 18443-81
Copyright: © 2012 S. Palchaudhuri et al.
Copyright year: 2012
License: This is an open-access article distributed under the terms of the Creative Commons Attribution License, which permits unrestricted use, distribution, and reproduction in any medium, provided the original work is properly cited.
License URL: http://creativecommons.org/licenses/by/2.0/

JOURNAL INFORMATION
==============================
NLM Title Abbreviation: J Int AIDS Soc
Journal ID: J Int AIDS Soc
NLM Journal ID: 101478566
Journal ID: JIAS

Journal of the International AIDS Society

EISSN: 1758-2652
Publisher: Wiley

ARTICLE INFORMATION
==============================
Article ID: 18443-82
Subjects: E82 - Operations research in the use of laboratory monitoring, other than viral load

TUPDE0204: Evaluating the effect of the use of point-of-care CD4 machines on access to antiretroviral therapy (ART) eligibility screening and ART initiation for HIV-positive pregnant women in Zimbabwe: towards elimination of new paediatric HIV infection by 2015

Muchedzi A. 1 §
Chadambuka A. 1
Chikwinya B. 1
Mahomva A. 1
1 Elizabeth Glaser Pediatric AIDS Foundation (EGPAF), Harare, Zimbabwe
§ Presenting author email: amuchedzi@pedaids.org
Electronic publication date: 2012 Oct 22
Publication date: 2012
Volume: 15
Issue: Suppl 3
Electronic Location ID: 18443-82
Copyright: © 2012 A. Muchedzi et al.
Copyright year: 2012
License: This is an open-access article distributed under the terms of the Creative Commons Attribution License, which permits unrestricted use, distribution, and reproduction in any medium, provided the original work is properly cited.
License URL: http://creativecommons.org/licenses/by/2.0/

JOURNAL INFORMATION
==============================
NLM Title Abbreviation: J Int AIDS Soc
Journal ID: J Int AIDS Soc
NLM Journal ID: 101478566
Journal ID: JIAS

Journal of the International AIDS Society

EISSN: 1758-2652
Publisher: Wiley

ARTICLE INFORMATION
==============================
Article ID: 18443-83
Subjects: E83 - International trade law and intellectual property

WEAD0405: Methodology to determine the patent status of key ARVs and other essential medicines: a strategy to reduce cost of treatment

Dhaliwal M. 1
Avafia T. 2
O'Malley J. 2
Konstantinov B. 1
Milani B. 3
Oh C. 4
UNDP HIV/AIDS Practice
1 United Nations Development Programme, BDP, HIV/AIDS Group, New York, United States
2 United Nations Development Programme, New York, United States
3 Medecins Sans Frontieres, New York, United States
4 Independent, New York, United States
Electronic publication date: 2012 Oct 22
Publication date: 2012
Volume: 15
Issue: Suppl 3
Electronic Location ID: 18443-83
Copyright: © 2012 M. Dhaliwal et al.
Copyright year: 2012
License: This is an open-access article distributed under the terms of the Creative Commons Attribution License, which permits unrestricted use, distribution, and reproduction in any medium, provided the original work is properly cited.
License URL: http://creativecommons.org/licenses/by/2.0/

JOURNAL INFORMATION
==============================
NLM Title Abbreviation: J Int AIDS Soc
Journal ID: J Int AIDS Soc
NLM Journal ID: 101478566
Journal ID: JIAS

Journal of the International AIDS Society

EISSN: 1758-2652
Publisher: Wiley

ARTICLE INFORMATION
==============================
Article ID: 18443-84
Subjects: E83 - International trade law and intellectual property

WEAE0103: Understanding voluntary licensing: an analysis of current practices and key provisions in antiretroviral voluntary licenses

Park C. 1 §
Moon S. 2
Burrone E. 1
Boulet P. 3
Juneja S. 1
‘t Hoen E. 1
1 Medicines Patent Pool, Geneva, Switzerland
2 Harvard University, Boston, United States
3 Independent Consultant, Geneva, Switzerland
§ Presenting author email: cpark@medicinespatentpoo.org
Electronic publication date: 2012 Oct 22
Publication date: 2012
Volume: 15
Issue: Suppl 3
Electronic Location ID: 18443-84
Copyright: © 2012 C. Park et al.
Copyright year: 2012
License: This is an open-access article distributed under the terms of the Creative Commons Attribution License, which permits unrestricted use, distribution, and reproduction in any medium, provided the original work is properly cited.
License URL: http://creativecommons.org/licenses/by/2.0/

JOURNAL INFORMATION
==============================
NLM Title Abbreviation: J Int AIDS Soc
Journal ID: J Int AIDS Soc
NLM Journal ID: 101478566
Journal ID: JIAS

Journal of the International AIDS Society

EISSN: 1758-2652
Publisher: Wiley

ARTICLE INFORMATION
==============================
Article ID: 18443-85
Subjects: E83 - International trade law and intellectual property

WEAE0104: Panorama of the pharmaceutical patenting and sanitary registration of ARVs drugs in Brazil: implications to access and to health industrial complex

Villardi P. 1 §
1 Associação Brasileira Interdisciplinar de AIDS, Rio de Janeiro, Brazil
§ Presenting author email: pedro@abiaids.org.br
Electronic publication date: 2012 Oct 22
Publication date: 2012
Volume: 15
Issue: Suppl 3
Electronic Location ID: 18443-85
Copyright: © 2012 P. Villardi et al.
Copyright year: 2012
License: This is an open-access article distributed under the terms of the Creative Commons Attribution License, which permits unrestricted use, distribution, and reproduction in any medium, provided the original work is properly cited.
License URL: http://creativecommons.org/licenses/by/2.0/

JOURNAL INFORMATION
==============================
NLM Title Abbreviation: J Int AIDS Soc
Journal ID: J Int AIDS Soc
NLM Journal ID: 101478566
Journal ID: JIAS

Journal of the International AIDS Society

EISSN: 1758-2652
Publisher: Wiley

ARTICLE INFORMATION
==============================
Article ID: 18443-86
Subjects: E85 - Role of multilateral agencies and donors

THPDD0105: Conflict-affected displaced persons need to benefit more from HIV Global Fund grants and national strategic plans

Spiegel P. 1 §
Hering H. 2
Paik E. 3
Schilperoord M. 1
1 UNHCR, Division of Programme Support and Management, Geneva, Switzerland
2 UNHCR, Cairo, Egypt
3 Independent Consultant, Paris, France
§ Presenting author email: spiegel@unhcr.org
Electronic publication date: 2012 Oct 22
Publication date: 2012
Volume: 15
Issue: Suppl 3
Electronic Location ID: 18443-86
Copyright: © 2012 P. Spiegel et al.
Copyright year: 2012
License: This is an open-access article distributed under the terms of the Creative Commons Attribution License, which permits unrestricted use, distribution, and reproduction in any medium, provided the original work is properly cited.
License URL: http://creativecommons.org/licenses/by/2.0/

JOURNAL INFORMATION
==============================
NLM Title Abbreviation: J Int AIDS Soc
Journal ID: J Int AIDS Soc
NLM Journal ID: 101478566
Journal ID: JIAS

Journal of the International AIDS Society

EISSN: 1758-2652
Publisher: Wiley

ARTICLE INFORMATION
==============================
Article ID: 18443-87
Subjects: E87 - International assistance and funding mechanisms

WEPDE0203: Its ‘dark ripple' effect: competing narratives of PEPFAR and sex work in southern Africa

Ditmore M. 1 §
Allman D. 2
1 Urban Justice Center Sex Workers Project, New York, United States
2 Dalla Lana School of Public Health, University of Toronto, Toronto, Canada
§ Presenting author email: mhd-iac@taumail.com
Electronic publication date: 2012 Oct 22
Publication date: 2012
Volume: 15
Issue: Suppl 3
Electronic Location ID: 18443-87
Copyright: © 2012 M. Ditmore et al.
Copyright year: 2012
License: This is an open-access article distributed under the terms of the Creative Commons Attribution License, which permits unrestricted use, distribution, and reproduction in any medium, provided the original work is properly cited.
License URL: http://creativecommons.org/licenses/by/2.0/

JOURNAL INFORMATION
==============================
NLM Title Abbreviation: J Int AIDS Soc
Journal ID: J Int AIDS Soc
NLM Journal ID: 101478566
Journal ID: JIAS

Journal of the International AIDS Society

EISSN: 1758-2652
Publisher: Wiley

ARTICLE INFORMATION
==============================
Article ID: 18443-88
Subjects: E89 - Financial sustainability of the response to HIV and AIDS

MOAE0304: Ensuring the financial sustainability of the national AIDS response in a low-middle income country with the growing HIV epidemic: is it feasible in the next few years in Ukraine?

Shakarishvili A. 1 §
Sharapka K. 1
Gotsadze G. 2
Ilnitski A. 1
Nizova N. 3
Zhyhinas O. 3
1 UNAIDS, Kiev, Ukraine
2 Curatio International Foundation, Tbilisi, Georgia
3 Ukrainian AIDS Centre of the Ministry of Health of Ukraine, Kiev, Ukraine
§ Presenting author email: shakarishvilia@unaids.org
Electronic publication date: 2012 Oct 22
Publication date: 2012
Volume: 15
Issue: Suppl 3
Electronic Location ID: 18443-88
Copyright: © 2012 A. Shakarishvili et al.
Copyright year: 2012
License: This is an open-access article distributed under the terms of the Creative Commons Attribution License, which permits unrestricted use, distribution, and reproduction in any medium, provided the original work is properly cited.
License URL: http://creativecommons.org/licenses/by/2.0/

JOURNAL INFORMATION
==============================
NLM Title Abbreviation: J Int AIDS Soc
Journal ID: J Int AIDS Soc
NLM Journal ID: 101478566
Journal ID: JIAS

Journal of the International AIDS Society

EISSN: 1758-2652
Publisher: Wiley

ARTICLE INFORMATION
==============================
Article ID: 18443-89
Subjects: E91 - AIDS: taking a long-term view and developing more positive and sustainable outcomes

WEPDE0104: Evaluating the costs of implementing a more sustainable response: a study of PEPFAR support to Kenya's PMTCT program, 2005–2010

Dutta A. 1 2
Wallace N. 1 2
Savosnick P. 3
Adungosi J. 4
Kioko U. 2 5
Stewart S. 6
Hijazi M. 6
Gichanga B. 7
1 Futures Group International LLC, Washington, United States
2 Health Policy Initiative Costing Task Order, Nairobi/Washington, Kenya
3 Elizabeth Glaser Pediatric AIDS Foundation, Nairobi, Kenya
4 FHI 360, Nairobi, Kenya
5 University of Nairobi, Nairobi, Kenya
6 Office of HIV/AIDS USAID, Washington, United States
7 USAID Kenya, Nairobi, Kenya
Electronic publication date: 2012 Oct 22
Publication date: 2012
Volume: 15
Issue: Suppl 3
Electronic Location ID: 18443-89
Copyright: © 2012 A. Dutta et al.
Copyright year: 2012
License: This is an open-access article distributed under the terms of the Creative Commons Attribution License, which permits unrestricted use, distribution, and reproduction in any medium, provided the original work is properly cited.
License URL: http://creativecommons.org/licenses/by/2.0/

JOURNAL INFORMATION
==============================
NLM Title Abbreviation: J Int AIDS Soc
Journal ID: J Int AIDS Soc
NLM Journal ID: 101478566
Journal ID: JIAS

Journal of the International AIDS Society

EISSN: 1758-2652
Publisher: Wiley

ARTICLE INFORMATION
==============================
Article ID: 18443-90
Subjects: E92 - Global recession and funding cutbacks: unleashing new challenges for sustainability

WEAE0106: The ‘middle-income’ curse: should global aid and treatment access decisions be based on national economic criteria?

Bhardwaj K. 1
1 Independent Lawyer (HIV, Health and Human Rights), New Delhi, India
Electronic publication date: 2012 Oct 22
Publication date: 2012
Volume: 15
Issue: Suppl 3
Electronic Location ID: 18443-90
Copyright: © 2012 K. Bhardwaj et al.
Copyright year: 2012
License: This is an open-access article distributed under the terms of the Creative Commons Attribution License, which permits unrestricted use, distribution, and reproduction in any medium, provided the original work is properly cited.
License URL: http://creativecommons.org/licenses/by/2.0/

JOURNAL INFORMATION
==============================
NLM Title Abbreviation: J Int AIDS Soc
Journal ID: J Int AIDS Soc
NLM Journal ID: 101478566
Journal ID: JIAS

Journal of the International AIDS Society

EISSN: 1758-2652
Publisher: Wiley

ARTICLE INFORMATION
==============================
Article ID: 18443-91
Subjects: E92 - Global recession and funding cutbacks: unleashing new challenges for sustainability

THPDE0204: Patient outcomes in Lubumbashi, Democratic Republic of Congo after disruption in HIV care and treatment due to decreased Global Fund appropriations

Freeman A. 1 §
Kiumbu M. 2
Mwamba B. 3
Atibu J. 2
Kalenga L. 3
Mukumbi H. 4
Ntambwe T. 3
Ramazani R. 3
Mafutamingi J. 3
Mwepu C. 3
Kalindula B. 3
Mbuya V. 3
Mwila L. 3
Cummiskey C. 5
Stolka K. 6
Newman J. 6
Hemingway-Foday J. 6
1 RTI International, Statistics and Epidemiology, Research Triangle Park, United States
2 Kinshasa School of Public Health, Kinshasa, Democratic Republic of the Congo
3 AMO-Congo, Lubumbashi, Democratic Republic of the Congo
4 AMO-Congo, Kinshasa, Democratic Republic of the Congo
5 RTI International, Washington, United States
6 RTI International, Research Triangle Park, United States
§ Presenting author email: afreeman@rti.org
Electronic publication date: 2012 Oct 22
Publication date: 2012
Volume: 15
Issue: Suppl 3
Electronic Location ID: 18443-91
Copyright: © 2012 A. Freeman et al.
Copyright year: 2012
License: This is an open-access article distributed under the terms of the Creative Commons Attribution License, which permits unrestricted use, distribution, and reproduction in any medium, provided the original work is properly cited.
License URL: http://creativecommons.org/licenses/by/2.0/

